# How Does LC/MS Compare to UV in Coffee Authentication and Determination of Antioxidant Effects? Brazilian and Middle Eastern Coffee as Case Studies

**DOI:** 10.3390/antiox11010131

**Published:** 2022-01-07

**Authors:** Enas A. El-Hawary, Ahmed Zayed, Annegret Laub, Luzia V. Modolo, Ludger Wessjohann, Mohamed A. Farag

**Affiliations:** 1 Chemistry Department, School of Sciences & Engineering, The American University in Cairo, New Cairo 11835, Egypt; enaselhawary17@aucegypt.edu; 2 Pharmacognosy Department, College of Pharmacy, Tanta University, Elguish Street (Medical Campus), Tanta 31527, Egypt; ahmed.zayed1@pharm.tanta.edu.eg; 3 Institute of Bioprocess Engineering, Technical University of Kaiserslautern, Gottlieb-Daimler-Straße 49, 67663 Kaiserslautern, Germany; 4 Department of Bioorganic Chemistry, Leibniz Institute of Plant Biochemistry, Weinberg 3, 06120 Halle, Germany; Annegret.Laub@ipb-halle.de; 5 Departamento de Botânica, Instituto de Ciências Biológicas, Universidade Federal de Minas Gerais, Belo Horizonte 31270-901, Brazil; vmodolo@icb.ufmg.br; 6 Pharmacognosy Department, College of Pharmacy, Cairo University, Kasr El-Aini St., Cairo 11562, Egypt

**Keywords:** antioxidant, chemometrics, chlorogenic acid, coffee, high-resolution mass spectrometry, UHPLC/MS, UV spectrometry

## Abstract

Coffee is a popular beverage owing to its unique flavor and diverse health benefits. The current study aimed at investigating the antioxidant activity, in relation to the phytochemical composition, of authenticated Brazilian green and roasted *Coffea arabica* and *C. robusta*, along with 15 commercial specimens collected from the Middle East. Ultra-high-performance liquid chromatography coupled to high-resolution mass spectrometry (UHPLC-ESI–HRMS) and UV spectrometry were employed for profiling and fingerprinting, respectively. With the aid of global natural product social molecular networking (GNPS), a total of 88 peaks were annotated as belonging to different chemical classes, of which 11 metabolites are reported for the first time in coffee seeds. Moreover, chemometric tools showed comparable results between both platforms, with more advantages for UV in the annotation of roasting products, suggesting that UV can serve as a discriminative tool. Additionally, antioxidant assays coupled with the UHPLC-ESI–HRMS dataset using partial least-squares discriminant analysis (PLS-DA) demonstrated that caffeoylquinic acid and caffeine were potential antioxidant markers in unroasted coffee versus dicaffeoyl quinolactone and melanoidins in roasted coffee. The study presents a multiplex metabolomics approach to the quality control of coffee, one of the most consumed beverages.

## 1. Introduction

Beverages containing caffeine, including coffee, are consumed daily worldwide to improve cognitive functions. Approximately three billion cups of coffee are consumed daily, expressed economically at the cost of ca. US $200 billion annually [1,2]. Though there are more than 120 species of *Coffea*, coffee is brewed mainly from the seeds of *Coffea arabica* L. and *C. canephora* L. var. *robusta* or *C. robusta* [3]. Arabica coffee is preferred by most consumers due to a more intense aroma and flavor compared to robusta [4].

Besides caffeine (1–4%), coffee seeds are rich in other secondary metabolites with several health benefits. Major secondary metabolites in coffee include phenolic acids, i.e., chlorogenic acids, which have various pharmacological properties, such as anti-inflammatory, hepatoprotective, anti-cancer, and anti-diabetic effects [5]. Moreover, coffee has been found to be enriched in diterpenes (0.7–3.5%), i.e., cafestol and kahweol, and other secondary metabolites [6]. In addition, the post-harvesting processing of coffee seeds, especially roasting, has been found to improve their sensory characters and to be associated with the production of roasting products, i.e., melanoidins. Furthermore, blending with aromatic herbs or spices is one of the most common traditions in the Middle East region to improve coffee aroma. Among the spices added to coffee, cardamom (*Elettaria cardamomum*) is considered a natural antioxidant and is used as a flavoring agent owing to its richness in volatiles, such as octyl acetate, terpineol, and terpinyl acetate. Other blends consisting of various aromatic herbs have been reported to be typical for certain regions of the Middle East as cardamom and Qassim blends. Produced by further processing of roasted coffee seeds, instant coffee products are often associated with a different metabolic profile affecting sensory attributes. They are also reported to be preferable for coffee consumers in the Middle East [7,8].

Synergism among several metabolites has been reported in plant extracts, including antioxidant activity in the case of coffee chemicals [9,10]. Hence, in-depth phytochemical analyses of bioactive metabolites using advanced analytical techniques, i.e., metabolomics, are warranted to identify active agents in such rich chemical matrixes. However, variation within the different species, the complexity of plant chemistries, and low levels of most secondary metabolites in plants warrant the development of sensitive analytical techniques to provide metabolome coverage [11].

Standardization and quality control of herbal products are major interests in food industry sectors, particularly methods based on fingerprinting. A typical platform for plant extract profiling includes chromatographic techniques, such as ultra-high-performance liquid chromatography coupled to high-resolution mass spectrometry (UHPLC-ESI–HRMS), which presents an excellent combination of separation and sensitivity with different modes of ionization [12,13]. Compared to UHPLC-ESI–HRMS, which enables the profiling of phytoextracts, UV spectrometry has been previously applied for the quantification of bioactive metabolites and the fingerprinting of herbal extracts [14] and coffee seeds as a fast, reliable, cheap, simple, and non-destructive technique [15,16], though with lower identification potential. With both analytical platforms, experimentalists are challenged with huge datasets, especially if derived from many samples, as is typical in quality control studies warranting analysis with chemometric tools for better data visualization and sample classification.

Continuing our previous work on coffee profiling, targeting aroma metabolites [8] and primary metabolites [17], we extend our research by reporting on the secondary metabolome in coffee, focusing, for the first time, on antioxidant properties. The current study provides a comprehensive analysis of 15 commercial coffee seeds collected from the Middle East region along with four authenticated green and roasted arabica and robusta coffee seeds. Specimen analyses were performed using and comparing two different platforms—UHPLC-ESI–HRMS and UV—with the aim of comparatively investigating the coffee metabolome for the first time in coffee analysis. In addition, global natural product social molecular networking (GNPS) coupled with UHPLC-ESI–HRMS enabled the annotation of 86 metabolites, 11 of which are for the first time reported in coffee seeds. Additionally, UV fingerprinting provided crucial absorption ranges (200–450 nm) of the different major metabolites—of potential use as an alternative tool for coffee quality control. Different in vitro antioxidant assays were conducted and found to be correlated with coffee metabolites, i.e., caffeoylquinic acid, dicaffeoyl quinolactone, and caffeine.

## 2. Materials and Methods

### 2.1. Samples Collection and Preparation

Authenticated green and roasted forms of both species of *Coffea*, i.e., *C. arabica* and *C. robusta* (four samples) were collected from Mina Gerais, Brazil, along with 15 commercial products representing different suppliers, roasting degrees, instant coffee, and blends well-known and preferred in the Middle East. All coffee specimens (19 samples) used in the current research, including their codes, are listed in Table 1. Some of the samples were lab-roasted, the roasting performed inside sealed glass vials for 6 h using a hot plate shaker SM 30 AT control (Edmund Bühler GmbH, Bodelshausen, Germany) set at 120 °C and with the shaking speed set at 150 rpm, following the method previously described by Abdelwareth et al [8]. Table 1 shows the roasting index of roasted coffee which was determined based on melanoidin levels. 

### 2.2. Samples Extraction and UHPLC-ESI–HRMS Analysis

Following the protocol previously developed by Farag et al. [18,19], with a few modifications, 150 mg of each coffee powder specimen was homogenized with 5 mL MeOH (100% *v/v*) containing 10 µg/mL umbelliferone as an internal standard using an Ultra-Turrax mixer (IKA, Staufen, Germany) adjusted at 11,000 rpm, five times for 20 s periods, with intervals of 1 min between each mixing period to guard against temperature increases and heating effects. The resultant suspensions were then vortexed vigorously, centrifuged at 3000× *g* for 30 min, and filtered through a 22 μm pore size filter to remove plant debris. Then, 1 mL was aliquoted and pre-treated by placement on a 500 mg C_18_ cartridge pre-conditioned with MeOH and Milli-Q water before elution, performed twice, using 3 mL MeOH. The eluent was afterwards evaporated under a nitrogen stream, and the obtained dry residue was re-suspended in 1 mL MeOH.

The principal step of UHPLC-ESI–HRMS analysis was conducted in triplicate (*n* = 3), with 2 µL introduced to an Dionex 3000 UHPLC system (Thermo Fisher Scientific, Bremen, Germany) equipped with a HSS T3 column (100 × 1.0 mm, 1.8 μm; Waters^®^; column temperature: 40 °C), and a photodiode array detector (PDA, Thermo Fisher Scientific, Bremen). The chromatographic conditions were optimized for improved peak elution using a binary gradient elution protocol at a flow rate of 150 μL/min. The composition of the mobile phase varied between water/formic acid, 99.9/0.1 (*v/v*) (A) and acetonitrile/formic acid 99.9/0.1 (*v/v*) (B). The protocol consisted, first, of an isocratic step for 1 min of 5% mobile phase B, then a linear increase of B from 5% to 100% over 11 min. The mobile phase was kept isocratic between 11–19 min at 100% B. Afterwards, there was a return to 5% B within 1 min, and, finally, an additional 10 min, i.e., 20–30 min, for column re-equilibration using 5% B. The wavelength range of the PDA measurements used for detection was 190–600 nm.

The UHPLC system was coupled with a high-resolution mass spectrometer using an Orbitrap Elite mass spectrometer (Thermo Fisher Scientific, Bremen, Germany) equipped with a HESI electrospray ion source (spray voltage: positive ion mode 4 kV, negative ion mode 3 kV; source heater temperature: 250 °C; capillary temperature: 300 °C; FTMS resolution: 30,000). Nitrogen was used as sheath and auxiliary gas. The CID mass spectra (buffer gas: helium; FTMS resolution: 15,000) were recorded in data-dependent acquisition mode (dda) using a normalized collision energy (NCE) of 35% and 45% The instrument was externally calibrated with Pierce^®^ LTQ Velos ESI positive ion calibration solution (product number 88323, Thermo Fisher Scientific, Rockford, IL, USA) and Pierce^®^ LTQ Velos ESI negative ion calibration solution (product number 88324, Thermo Fisher Scientific, Rockford, IL, USA).

### 2.3. UV Measurements and Multivariate Data Analysis

Three grams of each coffee sample was macerated with 30 mL methanol (100%) for 2 h, then centrifuged and filtered as previously described in Section 2.2. An aliquot of 200 µL was used, prepared from four replicates in a 96-well plate for the Gen 5 Greener UV microplate reader (Gen 5, kitted with a 96-well quartz cell with 1 nm spectral resolution in the UV region). Aliquots of each coffee extract (200 µL) were pipetted into the microplate wells (*n* = 4) of a Gen 5 Greener UV microplate reader (BioTek Instruments, Inc., Winooski, VT, USA). The absorption spectra were recorded in the range of 200–450 nm.

Afterwards, the spectral dataset was then converted to a data matrix using Excel (Excel 2010, Microsoft, Redmond, DC, USA). The matrix was constructed for all samples with their biological replicates for samples against 250 variables (wavelengths) spanning the readings. Finally, the dataset was subjected to unsupervised multivariate data analysis, including principal component analysis (PCA) using SIMCA software (version 14.1). All variables were mean-centered and pareto-scaled.

### 2.4. Tenative Identification of Metabolites Analyzed by UHPLC-ESI–HRMS

Metabolite identification was carried out based on retention times, accurate mass, fragments, comparison with reference standards, isotopic distribution, UV–Vis spectra, and errors reported in the literature and the Dictionary of Natural Products. The analysis was performed in both positive and negative modes, and the mass spectra derived from the protonated [M + H]^+^ or deprotonated [M − H]^−^ ions accompanied by their fragmentation patterns aided in structural elucidation. The chromatograms show components as functions of their retention time and mass-to-charge ratio by mass relative abundance. The high-resolution mass spectrometry data were evaluated with the Xcalibur software 2.2 SP1 (Thermo Fisher Scientific).

### 2.5. Molecular Based Networking of Coffee Specimens

Molecular networks were generated for negative ionization files applying Global Natural Products Social Molecular Networking (GNPS, https://gnps.ucsd.edu/ProteoSAFe/static/gnps-splash.jsp) accessed date (21 December 2020). For the building of networks, the following parameters were adjusted: 0.02 Da parent mass tolerance, 0.01 Da fragment ion tolerance, 0.7 or above cosine score, and a minimum of four matching peaks. In addition, cystoscope open-source software (version 3.8.2) was used for network visualization [20].

### 2.6. Determination of Total Phenolic Content

Total phenolic content (TPC) of the coffee specimen extracts, prepared as explained in Section 2.3, was determined calorimetrically using Folin–Ciocâlteu reagent as described by Zhang et al., with slight modifications, with gallic acid being used as a standard for quantification [21]. Briefly, 20 µL aliquots were mixed with 100 µL of 10% Folin–Ciocâlteu reagent and left for 5 min in the dark, followed by the addition of 80 µL 7.5 mg sodium bicarbonate and incubation in the dark for 30 min. The absorbance of all samples was measured at 765 nm. In addition, a standard curve of gallic acid was established in the concentration range of 1–100 µg/mL. All measurements were made in triplicate (*n* = 3) and the TPC was expressed as mg gallic acid equivalent/mg extract (mg GAE/mg extract).

### 2.7. Antioxidant Assays

#### 2.7.1. DPPH Radical Scavenging Assay

The DPPH radical scavenging assay was carried out as described by Hidalgo et al., with slight modifications [22]. Briefly, 30 µL of each extract prepared as described in Section 2.3 was mixed with 270 µL 6 × 10^−5^ M DPPH. The mixtures were then left in the dark for 30 min, and absorbance was recorded afterwards at 517 nm. A negative control sample was made of 30 µL 100% methanol instead of sample aliquots. All measurements were made in triplicate using a microplate reader at different concentrations, i.e., 0.01, 0.1, and 0.5 µg/mL, and the results were expressed as mean ± SD.

The radical scavenging activity was measured for each specimen as percentage inhibition of DPPH = (1 − A_s_/A_c_) × 100, where A_s_ stands for sample absorbance and A_c_ for negative control. The IC_50_ ± SD (µg/mL) values were then calculated, representing the percentage required by the samples to decrease DPPH absorption by 50%; therefore, higher IC_50_ values indicate the lower antioxidant activity of the coffee samples.

#### 2.7.2. Ferric Reducing Antioxidant Power (FRAP) Assay

The FRAP assay is also a colorimetric assay that measures the ferric reducing power of samples. According to Fernández-Poyatos et al.’s protocol, a FRAP assay was conducted. Briefly, 175 µL of freshly prepared FRAP reagent, consisting of 10 mM TPTZ (2,4,6-tripyridyl-*S*-triazine) in 40 mM HCl (10 Mm), acetate buffer (300 mM, pH 3.6), and 20 mM FeCl_3_, was mixed with 25 μL of extract and incubated in the dark for 30 min till recordings were made at 593 nm. A Trolox calibration curve (0.01–0.1 mg/mL) was constructed. The results were expressed as mg Trolox equivalents per mg extract (mg TE/g) [23]. All the measurements were performed in triplicate (*n* = 3) and expressed as mean ± SD.

## 3. Results and Discussion

The main objective of this study was to identify heterogeneity in coffee metabolites with reference to suppliers, roasting methods, and different commercial blends in the Middle East. To achieve this objective, methanol extracts prepared from authenticated and commercial coffee specimens were analyzed using UHPLC-ESI–HRMS and UV–Vis. In addition, total phenolic content (TPC) and one of the most important properties of coffee, viz., antioxidant activity, were determined in relation to the UHPLC-ESI–HRMS and UV–Vis datasets for comparison between both analytical platforms.

### 3.1. Metabolite Profiling via UHPLC-ESI–HRMS

Authenticated green and roasted coffee seeds, i.e., GCA, GCC, RCA, and RCC (Table 1), were first subjected to UHPLC-ESI–HRMS metabolite profiling. The results revealed qualitative and quantitative differences in the peaks detected in authenticated coffee seed extracts derived from both species, either in positive or negative mode (Supplementary Figure S1A,B). The metabolites were eluted in the order of organic acids, phenolic acid glycosides, alkaloids, hydroxycinnamic acids, diterpenoids, *N*-alkanoyl fatty acids, sphingolipids, and fatty acids. While the negative mode was able to detect most classes of metabolites, the positive mode was more suitable for alkaloids, diterpenes, amides, and nitrogenous compounds (see Table 2). Hence, both modes complemented each other, covering the identification of numerous compounds.

Furthermore, GNPS is a system that calculates the scores between all the fragment ions (MS/MS) inside a dataset as an early step in the launch of a molecular network to analyze sets of data in comparison with all public data. The molecular networks generated by GNPS are considered as a visual exhibition of a group of spectra of structurally related molecules. Each node represents a spectrum that provides information from a metadata file describing special properties of the supplemented files, such as sample species, processing, type, etc. On the other hand, the edges correspond to the alignment between spectrums, and connections between two nodes contributes to the formation of clusters of similar molecules known as molecular families that allow the user to distinguish between the distinct families included in the network. Finally, for the visualization of molecular networks, the data were imported into Cytoscape software for further analysis (gnps.ucsd.edu) [20]. GNPS has been applied successfully for various naturally derived extracts and could potentially identify a number of metabolites [47,48,49].

**Figure 1:**
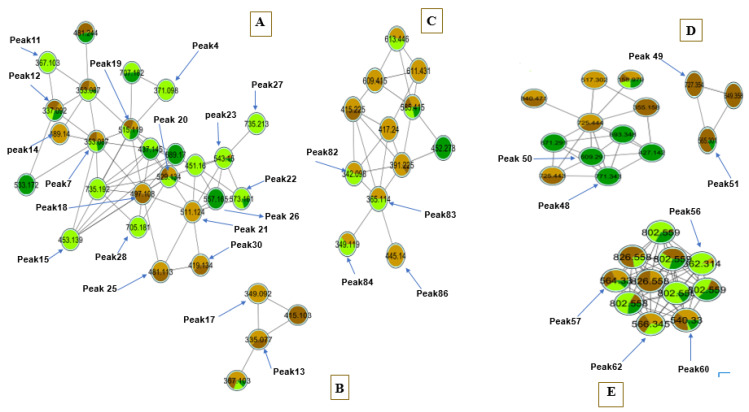
Molecular networks of major clusters created from authenticated coffee samples, i.e., GCA, GCC, RCA, and RCC. For all the networks, nodes are color-coded based on the roasting and species present and labelled by their parent ions. Light and dark green correspond to green *Coffea robusta* and green *C. arabica*, respectively, while light and dark brown correspond to roasted *C. robusta* and roasted *C. arabica*, respectively. (**A**) Molecular network of hydroxycinnamate esters. (**B**) Hydroxycinnamic lactones. (**C**) Hydroxycinnamic amides. (**D**) Diterpenes. (**E**) Fatty acids and sphingolipids.

GNPS was applied for the visualization of the coffee metabolome obtained from the UHPLC-ESI–HRMS platform for authenticated green and roasted coffee seeds [20]. The graphical display aided in the annotation and dereplication of the metabolites obtained from the UHPLC-ESI–HRMS datasets and in the tentative identification of unknown peaks. The molecular networking (MN) that was created encompassed 145 connected nodes consisting of 11 clusters, the nodes of the network representing the compounds’ parent ions and the colors of the node representing the roasting and species attributes provided from the metadata file (Figure 1A–E).

A total of 88 peaks were annotated as belonging to different metabolite classes, including hydroxycinnamic acid esters, lactones and amides (30 compounds), fatty acids and sphingolipids (**22**), diterpenes and diterpene glycosides (**17**), alkaloids (**2**), phenolic acid glycosides (**2**), in addition to other classes that were tentatively identified. Metabolite assignments were mostly based on tandem MS spectra showing unique fragmentation patterns for the metabolites (Supplementary Figures S2–S21), as explained in the next sub-sections for each class. Table 2 lists all spectral data for the identified peaks in coffee seeds of both species.

#### 3.1.1. Alkaloids

Two alkaloids were identified in peaks P5 and P6, annotated as trigonelline and caffeine, respectively, with a higher abundance of caffeine than trigonelline (Supplementary Figure S1B). Caffeine belongs to the xanthine alkaloids detected in the positive ion mode owing to the presence of a nitrogen atom (P6, 3.54 min) at *m/z* 195 for [M + H]^+^ and a yielding fragment ion at *m/z* 138 for the loss of methyl isocyanate (*m/z* 57) (Supplementary Figure S2). Trigonelline is a bitter alkaloid that contributes to coffee flavor and was detected at *m/z* 138 for [M + H]^+^ (P5, 1.37 min), showing a fragment ion at *m/z* 93 corresponding to a methyl pyridinium ion (Supplementary Figure S3 and Table 2) [26].

#### 3.1.2. Hydroxycinnamate Derivatives

Coffee seeds represent typically rich sources of hydroxycinnamic acid (HCA) derivatives in the form of major chlorogenic acids. These compounds included mono-acylquinic acids, i.e., mono-caffeoyl, mono-*p*-coumaroyl, and mono-feruloyl or diacyl, i.e., di-caffeoylquinic acid isomers, and tri-acyl, i.e., *O*-caffeoyl-*O*-feruloyl-*O*-sinapoylquinic acid [31].

Thirty HCA derivatives were identified, including acids, esters, lactones, and amides, among which dicaffeoyl quinic acid (P19) was the most abundant (Supplementary Figure S1A), identified via its [M − H]^−^ at *m/z* 515.11859 with fragment ions at *m/z* 353 [M-H-162]^−^ and 335 [M-H-180]^−^ for [caffeic acid-H_2_O]^−^ and caffeic acid losses, respectively (Supplementary Figure S4) [30]. Another less abundant peak (P20) than P19 resulted in *m/z* 529 [M − H]^−^ and showed fragment ions at *m/z* 367 and *m/z* 353 corresponding to the respective losses of [caffeic acid-H_2_O] and [ferulic acid-H_2_O] and was annotated as caffeoyl-feruloylquinic acid (Supplementary Figure S5). The compound annotations were consistent with the previous literature [25]. Both compounds were detected in the four types of authenticated seeds.

In agreement with the literature, several minor chlorogenic acid derivatives were also characterized in both green and roasted coffee seeds, i.e., P16, P22, and P28. For example, a peak at *m/z* 381 found to be more abundant in RCC than RCA was annotated as methyl-*O*-feruloyl quinate (P16) and confirmed from its fragment ions at *m/z* 175, [M-H-ferulic acid- H_2_O]^−^, *m/z* 193 [ferulic acid anion]^−^, and *m/z* 349 [M-feruloyl-quinolactone]^−^ (Supplementary Figure S6). Furthermore, a peak derived from sinapic acid was assigned to sinapoyl-feruloyl quinic acid (P22) at *m/z* 573 and fragment ions at *m/z* 397 [M-sinapoylquinic acid-H]^−^ and *m/z* 349 [M-feruloylquinic acid-H-H_2_O]^−^. P22 was previously reported in *C. robusta* and this is the first time it has been reported in green *arabica* coffee (GCA) (Supplementary Figure S7) [31].

Moreover, triacyl quinic acid derivatives were identified in several peaks and recognized as potential markers. For instance, P28 revealed a compound with a parent peak at *m/z* 705 for [M − H]^−^ and fragment ions at *m/z* 543 and 529, with the loss of a caffeoyl moiety annotated as di-*O*-feruloyl-*O*-caffeoylquinic acid (P28), a marker for green *robusta* coffee (GCC) being absent in *arabica* seeds (Supplementary Figure S8) [33]. Additionally, another triacyl quinic acid is reported here for the first time in GCC at *m/z* 735 [M − H]^−^, annotated as triacyl *O*-caffeoyl-*O*-feruloyl-*O*-sinapoylquinic acid in (P27), based on *m/z* 573 [M-caffeic acid-H_2_O]^−^ and *m/z* 529 [M-caffeic acid-H_2_O-CO_2_]^−^ fragment ions (Supplementary Figure S9). A tentative identification was further supported by the obtained molecular network (MN) which displayed structural similarity to di-*O*-feruloyl-*O*-caffeoylquinic acid (P28) (Figure 1A).

Furthermore, another novel marker for GCC was dihydroferulic acid-*O*-glucuronide (P4), which showed an [M − H]^−^ ion at *m/z* 371 and MS^2^ fragments at *m/z* 353, 191, 173, and 135. The loss of *m/z* 162 (hexose) directly connected to dicaffeoyl quinic acid in the MN suggested its phenolic acid glycoside constitution (Table 2, Figure 1B, and Supplementary Figure S10) [25].

Aside from hydroxycinnamic acid esters, lactones (quinides) were identified specifically in roasted seeds of both species, i.e., RCC and RCA, and hence considered as roasting-associated products. Their annotations were confirmed by their clustering together in the MN (Figure 1B). Examples included P17 with a molecular ion [M − H]^−^ at *m/z* 349 and MS^2^ fragments at *m/z* 175 [ferulic acid-H-H_2_O]^−^ and *m/z* 193 [ferulic acid-H]^−^, annotated as feruloyl quinolactone (Supplementary Figure S11) [32]. Lastly, five hydroxycinnamoyl amides were identified mostly in *robusta* coffee seeds, i.e., P82 and P83 in GCC and RCC (Table 2). The MN (Figure 1C) showed most of the identified hydroxycinnamoyl amides, as identified in Table 2, indicating their structural similarities. The results were in agreement with the previous literature distinguishing *C. robusta* products from other *Coffea* species-containing analogues [46].

#### 3.1.3. Diterpenes

The major diterpenes in coffee are cafestol and kahweol, reported in both *C. arabica* and *C. robusta* species [41]. In the current study, with the aid of MN, several diterpenes were identified in investigated coffee samples (Figure 1D). They were detected in positive ion mode given their lack of an electronegative group, as in phenolic compounds, highlighting the importance of profiling in different ionization modes [50]. For instance, cafestol (P35) was detected at *m/z* 317 [M + H]^+^, along with its dehydrated analogue (P37), assigned as dehydrocafestol at *m/z* 299 [M + H]^+^ (Supplementary Figures S12 and S13).

Additionally, a new diterpene was identified in P45, particularly in roasted seeds of both species, i.e., RCA and RCC. It has been reported for the first time in coffee seeds derived from dehydrocafestol by a further dehydration step which may easily occur during seed roasting and was annotated as a dehydrocafestol derivative. It was assigned based on *m/z* 281 (calculated for [C_20_H_25_O]^+^, *m/z* 281.1895) and corresponded to [M + H]^+^ and fragment ions at *m/z* 263 [M+H-H_2_O]^+^ and *m/z* 147 [M+H-C_10_H_18_O_2_]^+^, confirming the identity of the cafestol moiety (Supplementary Figure S14) [41]. Hence, such compounds may be recognized as roasting markers.

Moreover, mozambioside, a diterpenoid glycoside of furokaurane type, was reported previously as a marker for *C. arabica* species [39]. It was detected in the current study in both green and roasted *arabica* (P38) (Supplementary Figure S1B) at *m/z* 509 [M + H]^+^ with fragment ions at *m/z* 347 [M+H-hexose]^+^ and 329 [M+H-hexose-H_2_O]^+^ and with a characteristic UV band at λ_max_ 298 nm (Supplementary Figure S15) [39].

Interestingly, *m/z* 347 [M + H]^+^ in P36 corresponded to a new diterpenoid and was annotated as trihydroxy-kauradien-olide, based on fragment ions at *m/z* 329 [M+H-18]^+^ and *m/z* 285 [M+H-44]^+^ [38], presenting a new marker for green *arabica* species (GCA), since it was completely absent from roasted RCA seeds, which suggests its degradation upon roasting. P36 was identified in *Isodon* species and this is the first time it has been reported in coffee seeds (Supplementary Figure S16) [38].

#### 3.1.4. Fatty Acids and Sphingolipids

Seeds are well-known with their richness in lipids. Coffee seed analysis revealed that various fatty acids and sphingolipids appeared late at R_t_ > 12.00 min of the UHPLC chromatograms given their relatively nonpolar nature (Table 2). The negative ionization mode revealed several non-hydroxylated, i.e., P54 and P69, and hydroxylated fatty acid, i.e., P52, P71, and P72, peaks. An example of a non-hydroxylated fatty acid was P69 at *m/z* 341 [M − H]^−^ assigned as dimethyl octadecanedioate, with fragment ions at *m/z* 313 [M-H-2CH_2_]^−^ and *m/z* 269 [M-H-2CH_2_-COO]^−^ (Supplementary Figure S17). P69 was detected in all investigated coffee samples. In addition, the hydroxylated fatty acids in P52 showed [M − H]^−^ at *m/z* 329, 483, and 355, respectively. They were annotated with reference to the literature [11]. It is worth mentioning that P52, i.e., trihydroxy-octadecaenoic acid, was only detected in green and roasted arabica coffee (Table 2).

Nitrogen-containing lipids, including various sphingolipids and fatty acyl amides, were also detected. In particular, P74 *m/z* 338 [M + H]^+^ was annotated as docosenamide, which is a fatty acyl amide showing MS/MS fragments at *m/z* 321 [M+H-17]^+^, corresponding to the loss of ammonia, which is in agreement with previous literature [43]. It was detected in both roasted arabica and robusta seeds for the first time (Table 2 and Supplementary Figure S18). Therefore, P74 may be considered a roasting marker. The MN for fatty acids and sphingolipids is illustrated in Figure 1E.

#### 3.1.5. Serotonin Amides (Hydroxytryptamine Derivatives)

*N*-alkanoyl-5-hydroxytryptamides (C-5HTs) or serotonin amides are well documented in coffee seeds [44,45]. Several peaks corresponding to C-5HTs were detected in the positive mode, in which the amino group of the 5-hydroxytryptamine (5-HT) was acylated with an octadecanoyl (C18), eicosanoyl (C20), and heneicosanoyl (C21), yielding *N*-octadecanoyl-5-hydroxytryptamide (C18-5HT, P78), *N*-eicosanoyl-5-hydroxytryptamide (EHT, P79), and *N*-heneicosanoyl-5-hydroxytryptamide (C21-5HT, P75), respectively. These compounds were detected at *m/z* 443 [M + H]^+^ for C18 5-HT, *m/z* 471 for EHT, and *m/z* 487 for C21-5HT (Table 2). Moreover, *N*-tricosanoyl-5-hydroxytryptamine (C23-5HT, P76), *N*-docosanoyl-5-hydroxytryptamine (C22-5HT, P77), and *N*-tetracosanoyl-5-hydroxytryptamine (C24-5HT, P80) were detected at *m/z* 515, 499, and 527 [M + H]^+^, respectively. All compounds shared the same fragment ion at *m/z* 160, corresponding to 5-hydroxy tryptamine, confirmed by their UV λ_max_ at 226 nm detected in both green and roasted samples of coffee seeds, in agreement with previous reports (Supplementary Figures S19 and S20) [44,45]. C-5HTs have been reported as present in the insoluble phase of the waxy surface of coffee seeds and as possessing antinociceptive and anti-inflammatory effects [51]. However, these compounds were reported to have an irritant effect on the stomach mucosa in high doses, manifesting in gastric discomfort. So, the waxy layer has increasingly been removed through steaming and dewaxing [44,45].

#### 3.1.6. Miscellaneous

Phenylpropanoid esters of sucrose are common secondary metabolites in *Planta*. They are considered potential candidates for drug discovery [36]. However, they have not been reported before in coffee seeds. Feruloyl ester of sucrose, identified at *m/z* 735 (P33) in negative mode with a product ion at *m/z* 367, was detected in green *arabica* and *robusta* species and annotated as acetyl-diferuloyl sucrose (Supplementary Figure S21) [35].

### 3.2. UHPLC–HRMS Based Multivariate Data Analyses and Fingerprinting of Coffee Samples

Although differences in metabolite composition could be revealed from the visual inspection of the UHPLC–MS chromatograms of coffee specimens (Supplementary Figure S1), the dataset was holistically extracted from the UHPLC–HRMS using multivariate data analyses, especially considering the large number of samples (57 samples) represented by three biological replicates each. Several models were constructed to classify coffee samples, stratifying them according to their species, roasting indices, and different blends common in the Middle East, i.e., cardamom and Qassim, as discussed in the next subsections.

#### 3.2.1. Roasted versus Green Coffee

Based on the UHPLC–HRMS dataset, a PCA score plot was firstly applied unsupervised for the 19 coffee samples, including authenticated and commercial preparations, different symbols denoting roasted, i.e., RCA, RCC, LRCM, LRS, HRKC, BRK, LRCK, BRA, LRSQ, LRCS, LRCQ, ICA, ICC, versus green or unroasted ones, i.e., GCA, GCC, GCU, GCE, GCS, GCK. The PCA score plot (Supplementary Figure S22A) showed values for R^2^ = 0.49 and Q^2^ = 0.38, indicating an acceptable model, though not showing clear segregation of roasted from green samples, with some overlap between investigated specimens. A few markers appeared in the PCA loading plot (Supplementary Figure S22B), responsible for such segregation, including caffeine (P6) and, to a lesser extent, trigonelline (P5), which were enriched in roasted samples, while chlorogenic acid isomers, such as feruloyl quinic acid (P11), were found to be abundant in unroasted specimens.

**Figure 2:**
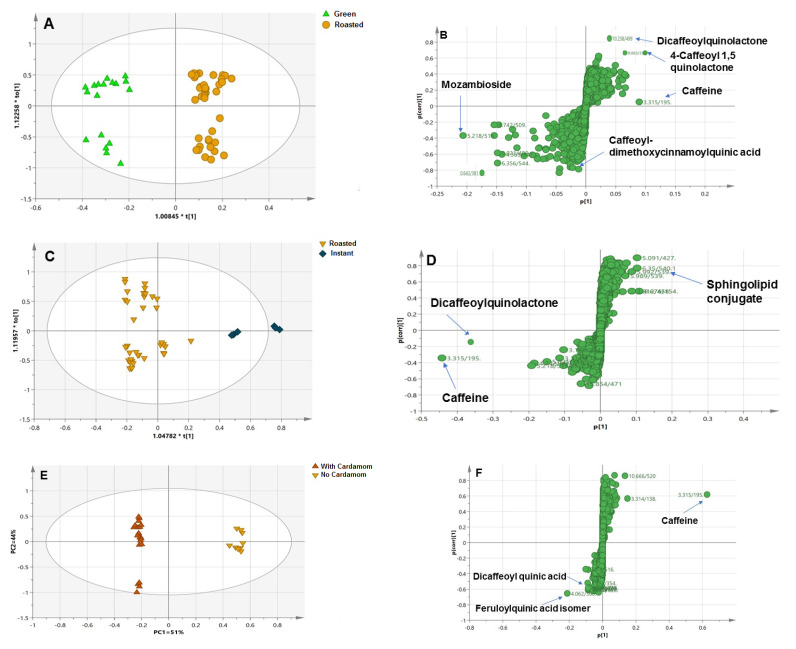
(**A**) OPLS-DA score plot based UPLC–MS analysis exploring green versus roasted coffee seeds. (**B**) OPLS-DA S-plot showing potential markers for green versus roasted coffee seeds. (**C**) OPLS-DA score plot for roasted versus instant samples. (**D**) OPLS-DA S-plot for roasted versus instant samples. (**E**) OPLS-DA score plot for plain versus blended coffee with cardamom samples. (**F**) OPLS-DA S-plot for plain versus blended coffee with cardamom samples. OPLS-DA S-plot models show covariance p [1] against the correlation p(cor) [1] for the variables of the discriminating components of the OPLS-DA models. Selected variables are highlighted in the S-plot and discussed in the text.

In another attempt to identify better markers and improve the classification potential of roasted versus unroasted samples, a supervised OPLS model was established for the same dataset. The supervised model showed R^2^ and Q^2^ of 0.91 and 0.83, respectively, with better sample segregation (Figure 2A). The S-plot showed other markers for HCAs, i.e., mozambioside (P38) and caffeoyl-dimethoxy cinnamoyl quinic acid (P24) for green specimens. In contrast, caffeoyl-quinolactone (P13) and dicaffeoyl-quinolactone (P18) were characteristic for the roasted samples and likely to be generated upon roasting as a result of dehydration reactions for the quinic acid derivatives and the formation of chlorogenic acid lactones (CGLs) (Figure 2B) [32].

#### 3.2.2. Instant versus Roasted Coffee Samples

As instant coffee production usually involves processing steps additional to roasting, PCA was employed to determine the variability between roasted and instant coffee samples (Supplementary Figure S22C). PCA was employed for 12 coffee samples, including an instant sample (ICA), an instant sample with cardamom (ICC), along with 10 roasted specimens denoted by different symbols (RCA, RCC, LRCM, LRS, HRKC, BRK, LRCK, BRA, LRSQ, LRCS). The PCA model revealed that the plain instant sample, i.e., ICA, was well separated from the other samples in the two PC projections, representing 40% of the total variance. The low PC values might be attributed to the fact that the instant sample blended with cardamom, i.e., ICC, was not segregated from the plain instant sample in addition to variation in the roasting degree of the coffee samples.

Consequently, an OPLS model was constructed, which showed better separation parameters (PC1 and PC2 = 85%). In addition, R^2^ and Q^2^ took the values 0.98 and 0.95, respectively (Figure 2C). The S-plot revealed that caffeine (P6) and dicaffeoyl quinolactone (P19) were the major markers for roasted samples, while sphingolipid conjugates, i.e., P60, were, interestingly, predicted to be the main markers for the instant sample (Figure 2D).

Another chemical that should have contributed to the separation of instant samples from roasted samples is acrylamide enrichment in instant as opposed to roasted coffee that has been reported in many studies [52]. However, it was hardly to be detected by LC–MS using the method employed in the current research. This may be attributed to its low molecular mass and concentrations better suited for detection by GC–MS or LC–MS after a clean-up pretreatment [52,53], suggesting that it would be of value to use more than one technique in metabolomics [18].

#### 3.2.3. Blended versus Plain Samples

To determine the impact of blending coffee as is typical in the Middle East region, PCA was employed to model 10 samples, including cardamom blended and plain samples, with specimens denoted by different symbols for cardamom and Qassim blended (LRCM, HRKC, LRCK, LRSQ, LRCS) versus plain ones (RCA, RCC, BRA, BRK, LRS). The score plot model showed the clustering of samples blended with cardamom mainly on the left side, while plain samples along with a few cardamom blended samples were placed on the right side along PC1 to cover 33% of the total variance (Supplementary Figure S22D). The loading plot demonstrated the possible enrichment of caffeine (P6) in plain coffee, while dicaffeoyl quinic acid (P19) and feruloyl quinic acid (P11) were found in blended products (Supplementary Figure S22E).

The OPLS score plot showed improved discrimination between investigated samples, with better PCs responsible for 91% of the total variance (R^2^ = 0.98 and Q^2^ = 0.91) (Figure 2E). Finally, the S-plot model confirmed PCA loading results regarding the higher abundance of caffeine in plain coffee versus chlorogenic acids, i.e., dicaffeoyl quinic acid and feruloyl quinic acid enrichment in coffee blended with cardamom products (Figure 2F). The obtained results were in accordance with a previous analysis of cardamom by HPLC which revealed its richness in phenolic compounds, i.e., tannic, caffeic, gallic, and dicaffeoyl quinic acids [54].

OPLS-DA model validation was assessed by the diagnostic metrics R^2^ (total variance) and Q^2^ (goodness parameters), which were greater than 0.8, with most models showing a regression line crossing zero, and with negative Q^2^ and R^2^ close to 1, signifying the model’s validation. Moreover, the *p*-values for each model were calculated using CV-ANOVA (analysis of variance of cross-validated residuals) and were all below 0.005 (Supplementary Figures S23–S25).

### 3.3. UV–Vis Fingerprinting of Coffee Seeds

The UV–Vis technique was further applied to compare models derived from UHPLC–HRMS based on metabolome analysis in coffee samples using the same extraction method employed for UHPLC–HRMS as a fingerprinting method. UV spectral bands provide information mostly about conjugated bioactive component characteristic profiles, i.e., phenolic acids (220–325 nm), methylxanthines (244–300 nm), and chlorogenic acids (200–325 nm). In this study, UV–Vis fingerprinting coupled to multivariate analysis was applied for differentiation between coffee specimens based on different variables examined with UHPLC–HRMS, i.e., genotypes, suppliers, roasting levels, and blending.

#### 3.3.1. Roasted versus Green Coffee Specimens

PCA modeling of the roasted and unroasted sample score plot successfully classified most of the samples along PC1 (91%) (Supplementary Figure S26A). Moreover, the loading line plot revealed intense absorption by green samples in the region of 220–350 nm, suggesting their richness in phenolic acids. In contrast, roasted samples were segregated based on their higher absorption values between 375 and 450 nm, which could be explained by the brown color factor or presence of melanoidins (λ_max_ = 420 nm). Melanoidins have been reported to exhibit different absorption bands in UV during roasting, early roasting (λ_max_ = 280 nm), medium roasting (λ_max_ = 330), and final roasting (λ_max_ = 420 nm). Hence, UV is suggested to be used for determining the roasting index in coffee for the coffee industry as a simple robust and inexpensive method compared to UHPLC–HRMS.

**Figure 3:**
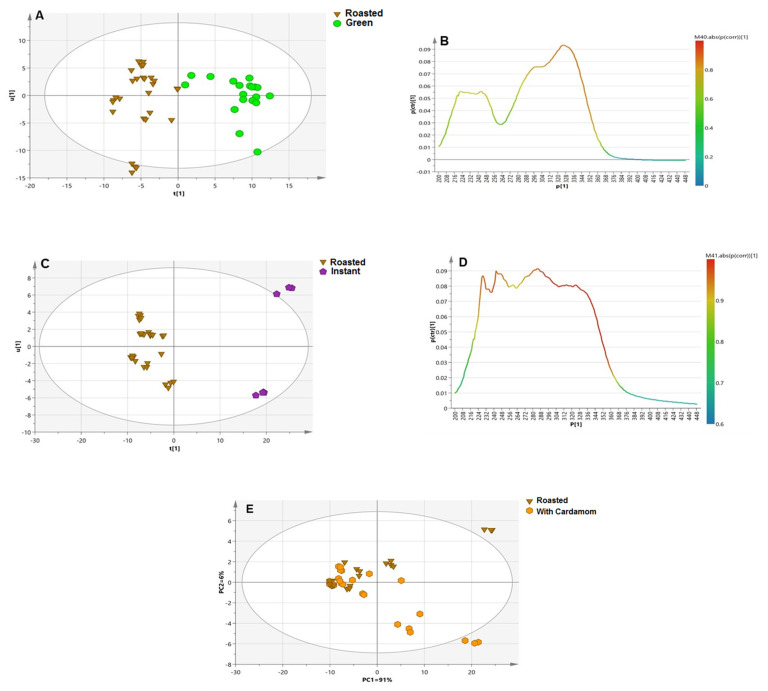
(**A**) OPLS-DA model for coffee samples (green versus roasted) based on UV–Vis analysis. (**B**) S-line based on roasting effect. (**C**) OPLS-DA for roasted versus instant samples. (**D**) S-line for instant and roasted samples. (**E**) PCA score plot model for plain roasted versus blended with cardamom samples. The S-plot shows the correlation (cor) and covariance p [1] between variables (wavelengths) and the predictive score of the discriminating component of OPLS-DA. The discriminant wavelengths in the important variables list are highlighted and discussed in the text.

Moreover, the OPLS-DA model was constructed for maximal separation of the samples. Green samples were distinctly clustered away from roasted samples and showed good validation parameters (R^2^ and Q^2^ = 1) (Figure 3A). Inspection of the corresponding S-line plot revealed that the discriminant wavelengths of green samples from roasted were at 210–230 nm and 300–330 nm, which is attributable to the absorption of phenolic acids (Figure 3B). These results were synchronous with those obtained from the S-plot for the UHPLC–MS model that revealed the enrichment of green coffee with chlorogenic acids (Figure 2B).

#### 3.3.2. Instant versus Roasted Samples

The instant samples, i.e., ICA and ICC, were well separated along PC1 (93%), appearing as outliers in the upper part of the plot (Supplementary Figure S26B). The corresponding loading plot revealed that instant samples absorbed more at 220 nm and 290 nm, which is likely attributable to fatty acids and sphingolipid conjugates, in accordance with UHPLC–HRMS results (Figure 2D). Interestingly, a UV λ_max_ at 275 nm was detected 3.5-fold compared to the roasted arabica sample, which is likely attributable to acrylamide, suggesting that instant coffee contains more acrylamide than roasted coffee, consistent with previous literature [52]. These results highlighted the way in which UV complemented results derived from UHPLC–MS by revealing potential coffee markers not detected in the later technique, including melanoidins and acrylamide, indicating coffee processing levels and further safety.

To confirm the acrylamide-derived band in UV, the spiking method with an acrylamide reference standard was attempted. The results showed that the absorbance of the sample spiked with acrylamide increased at 273 nm, in agreement with Alfarhani [55] (Supplementary Figure S27).

For further confirmation, an OPLS-DA model was built for better differentiation between the two sets of samples with R^2^ and Q^2^ values computed to be 0.97 and 0.91, respectively (Figure 3C). Upon investigation of the S-line plot of the OPLS-DA loading plot, characteristic UV regions of instant samples relative to roasted samples were identified in the range of 220–290 nm, likely due to the absorption wavelengths of sphingolipids (λ_max_ = 230 nm) and/or acrylamide (Figure 3D) [52].

#### 3.3.3. Blended versus Plain Coffee Samples

The PCA model was constructed to distinguish blended and plain coffee samples, with no clear segregation of cardamom and Qassim blended coffee samples from roasted samples and with overlap between the two specimens (Figure 3E). The loading plot showed the absorption of plain samples in the range of 350–450 nm, suggesting higher melanoidin levels, while cardamom blended samples, along with lightly roasted samples, were more rich in phenolic acids, with an absorption range of 220–350 nm, indicating cumulative phenolic content for both cardamom and roasted coffee [54].

### 3.4. Comparison between UHPLC–MS and UV Fingerprinting Multivariate Data Analysis Models

The classification potential of both UV and UHPLC–MS were compared based on their PCA and OPLS results. Both techniques were found generally comparable and to complement metabolite detection in the different coffee specimens. The PCA and OPLS loading plots obtained from UHPLC–MS revealed that caffeine and CGLs contributed to the discrimination of roasted samples compared with the abundance of chlorogenic acids and diterpenes in unroasted samples. In contrast, the interference of caffeine UV bands with chlorogenic acids could be predicted in UV models, albeit with roasted samples, to show tight clustering at higher absorption ranges, i.e., 350–450 nm, attributable to melanoidin absorption (λ_max_ = 420 nm) and not detected using UHPLC–MS. Other chemicals inferred from UV models were acrylamides, showing increased absorption in roasted specimens (Figure 3B). On the other hand, unroasted coffee samples showed higher absorbance in the region of 220–350 nm, typically for chlorogenic acid absorption (λ_max_ = 220 and 325 nm) and diterpenes (λ_max_ = 298 nm) (Figure 3B).

Likewise, the addition of cardamom to coffee was investigated by both techniques; the PCA score plot obtained from the UHPLC–MS measurements showed tighter clustering of instant samples than roasted samples, while the UV model could not distinguish clearly between roasted and cardamom blended samples, suggesting that blending effects are better revealed using UPLC–MS compared to a UV model.

However, the UHPLC–HRMS-derived model was neither able to detect acrylamide nor melanoidins that are important markers of processing impact on coffee and its safety. Nevertheless, the UV model loading plot showed that instant samples had strong absorption for acrylamide (λ_max_ = 275 nm), while roasted samples had a higher absorbance range of 370–420 nm, suggesting variation in melanoidin formation during the roasting technique. Accordingly, instant coffee is considered less safe than other types of coffee (Figure 2 and Figure 3). Comparative toxicological studies in animals should be pursued in the future to confirm such hypotheses generated using chemicals analyses.

### 3.5. Determination of Total Phenolic Content of Coffee Species

UHPLC–MS analysis revealed phenolic enrichment in coffee seeds (Table 2) to be affected by roasting [56]. Therefore, quantitative determinations of total phenolics were investigated in both commercial and authenticated coffee specimens and correlated with coffee seeds’ bioactivities, i.e., antioxidant action.

**Figure 4:**
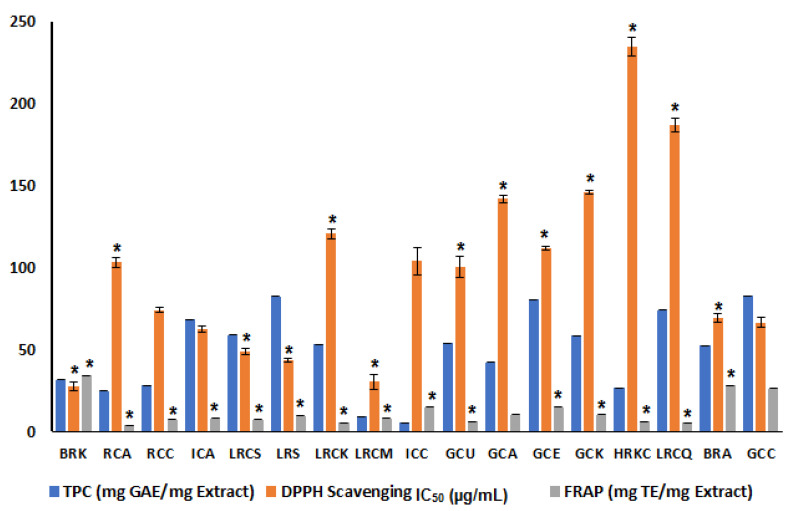
Total phenolic content (TPC) of the investigated coffee specimens with values expressed as mg gallic acid equivalent/g extract (mg GAE/g extract), DPPH (2,2-diphenyl-1-picrylhydrazyl) scavenging activity with values in IC_50_ ± SD (µg/mL), and ferric reducing antioxidant power (FRAP) with values expressed in mg Trolox equivalent per mg extract (mg TE/mg extract). Each bar represents mean ± SD (*n* = 3); the corresponding coffee specimen codes are listed in Table 1. *: Significant values compared to GCC specimen (*p* < 0.05).

The limit of detection (LOD) and limit of quantitation (LOQ) were calculated for the applied assay as 0.37 and 1.14 mg GAE/mg extract, respectively. The results of TPC showed that the highest levels were detected at 50–52 mg GAE/g in BRA, LRCK, and GCK, while the lowest were ICC and ICA at 3–7.7 mg GAE/g (Supplementary Table S1). In addition, increasing the roasting degree led to a decrease in TPC, with the highest level observed in lightly roasted samples and green samples and a marked decline in heavily roasted and instant samples. These findings were consistent with previous reports suggesting the superiority of green and light roasted coffee as a rich source of free polyphenols compared to processed (instant) and roasted coffee [57]. Nevertheless, differences between roasted samples may be attributed to the degradation of chlorogenic acids and their contribution to the development of Maillard reaction products, i.e., melanoidins [58]. Additionally, Arabian coffee blended with cardamom (ICC) and instant *C. arabica* (ICA) were recognized as having the lowest levels of phenolics, suggesting their degradation during the further processing steps and that instant coffee provides low phenolic levels, even compared to roasted coffee (Figure 4).

### 3.6. In Vitro Antioxidant Activity

#### 3.6.1. DPPH Assay

All coffee extracts displayed a dose-dependent DPPH radical scavenging activity in the concentration range, i.e., 0.01–0.5 µg/mL, with results expressed as IC_50_ (µg/mL) and R^2^ = 0.58. The DPPH IC_50_ values ranged from 27.3 µg/mL in lightly roasted *C. arabica* (BRK) up to 235.9 µg/mL in the heavily roasted *C. arabica* blended with cardamom (HRKC), compared to Trolox (IC_50_ = 12.4 µg/mL). The highest antioxidant capacity in lightly roasted coffee is consistent with previous reports [16]. The highest IC_50_ values in HRKC (235.9 µg/mL) and LRCQ (187 µg/mL) pointed out the low antioxidant potential in blended coffee and its correlation with TPC content (Supplementary Table S1).

In contrast, lightly roasted samples, including BRK, LRS, and LRCS, showed lower IC_50_ values, at 27.3, 43.2 and 48.6 µg/mL, respectively, suggesting their potential antioxidant power and that phenolics are more crucial than melanoidins for determining antioxidant action. A few roasted samples, such as RCC and RCA, had IC_50_ values at 74.2 and 103.3 µg/mL, respectively, indicating improvements in antioxidant activity that may be attributable to the production of melanoidins (Table 1 and Figure 4) [16].

#### 3.6.2. In Vitro FRAP Assay

To confirm results derived from DPPH, another antioxidant FRAP assay was examined. FRAP results were generally in accordance with the DPPH radical scavenging activity (Supplementary Table S1). Both BRK, BRA, and GCC samples showed the strongest antioxidant effect with FRAP values of 34.1, 28.2, and 26.19 mg TE/mg extract, respectively. In contrast, heavily roasted and instant samples HRKC, RCA, and ICC showed FRAP results at 6.3, 3.9, and 1.5 mg TE/mg extract, respectively. Cardamom addition in the different coffee blends did not result in an increase in FRAP values, as in HRKC (6.3 mg TE/mg extract), LRCM (7.9 mg TE/mg extract), and instant coffee products, i.e., ICC at 1.5 mg TE/mg extract, in accordance with the DPPH assay (Figure 4 and Supplementary Table S1).

### 3.7. Correlation between Biological Assays and UHPLC–MS Metabolite Profile

A correlation between the biological assays, i.e., the antioxidant and UHPLC–MS datasets, were attempted to determine the metabolites responsible for the antioxidant activity of the different coffee samples. Hence, a partial least-squares (PLS) model was constructed, taking the UHPLC–MS metabolites as x-variables and the corresponding biological assay parameters (TPC, DPPH, FRAP) as y-variables. The PLS score plot explained 99% of the total variance in Y (R^2^ = 0.99 and Q^2^ = 0.92) and as a prediction parameter explained 92%, the loading plot displaying a positive correlation with all assays. Investigation of variable importance in projection (VIP) enabled recognition of the metabolites responsible for the antioxidant effects and the pinpointing of the relation between the x- and y-variables in the PLS model. The main potential metabolites that had significant VIP scores included caffeine and caffeoylquinic acid, with VIP scores of 6.6 and 6.8, respectively.

The abundance of chlorogenic acid and its derivatives in coffee (7–12%), i.e., caffeoylquinic acids, resulted in additional potential antioxidant activities, including alleviation of cellular oxidative modulation. Additionally, several studies on caffeine revealed that it exerted hydrophilic and lipophilic antioxidant activity. Interestingly, dicaffeoyl quinolactone which is formed mainly during the roasting process, showed a lower score at 1.9, suggesting that it has a lower correlation potential and a lesser antioxidant effect, as demonstrated in the radar plot (Supplementary Figure S28).

## 4. Conclusions

The study represented a multiplex metabolomics approach using two different platforms—UHPLC–HRMS and UV fingerprinting techniques—for the tentative identification of secondary metabolites in different coffee products. Specimens differed with respect to several variables, such as genotype, roasting process, supplier, and additives. Both UHPLC–HRMS and UV spectroscopy coupled to multivariate data analysis revealed differences among authenticated Brazilian and commercial samples consumed in the Middle East. Such a comparative metabolomics approach presented the first detailed profile for green and roasted coffee metabolomes in that region. Additionally, GNPS aided in the identification of metabolites via UHPLC–HRMS data analysis, resulting in the tentative identification of several novel phenolics and diterpenes in coffee seeds. In contrast, UV fingerprinting provided preliminary data on the absorption ranges of the various main chemicals, showing its use as an alternative tool for UHPLC–HRMS, being cheaper and simpler to operate. Interestingly, both techniques were generally comparable with respect to metabolite detection in specimens, with more advantages found for UV in the identification of acrylamide and melanoidins—factors indicative of processing level. The developed comparative metabolomics approach can be considered for future quality control purposes in addition to other spectroscopic techniques. We have highlighted in the text that for unknown derivatives belonging to certain classes revealed by GNPS to have characteristic fragments/patterns, other spectral analyses, including NMR, should be considered in future work.

Furthermore, in vitro antioxidant assays provided a measure of how the antioxidant activity of different roasted and green coffee samples correlated with differences in metabolite composition, where phenolic compounds, such as caffeoylquinic acid, were more crucial than melanoidins. In addition, dicaffeoyl quinolactone had a lower impact on the antioxidant effect of coffee products compared to dicaffeoylquinic acid.

## Figures and Tables

**Table 1:** A list of coffee specimens analyzed using UHPLC-ESI–HRMS and UV spectroscopy, including origin, degree of roasting, and the sample code used in the text.

Sample Name		Supplier	Degree of Roasting *	Sample Code
Authentic roasted samples	Roasted *C. arabica*	Mina Gerais, Brazil	12.9	RCA
	Roasted *C. canephora* or *C. robusta*		6.6	RCC
Authentic green samples	Green *C. arabica*		7.3	GCA
	Green *C. canephora* or *C. robusta*		1.6	GCC
Commercial samples	Lightly roasted blended with cardamom	Maatouk, Saudi Arabia	1.8	LRCM
	Lightly roasted coffee	Shahi, Saudi Arabia	2.8	LRS
	Heavily roasted blended with cardamom	Alameed coffee, Kuwait	6.0	HRKC
	Lab-roasted green coffee		1.6	BRK
	Lightly roasted blended with cardamom		1.0	LRCK
	Lab-roasted green coffee	Aswan, Egypt	1.0	BRA
	Lightly roasted blended with Qassim blend	Saudi Arabia	figure--- **	LRSQ
	Lightly roasted blended with cardamom	Shahi, Saudi Arabia	4.1	LRCS
		Qatar	4.2	LRCQ
	Instant *C. arabica*	Maxima coffee	32.3	ICA
	Instant Arabian coffee blended with cardamom	NESCAFE Arabiana	7.1	ICC
	Green coffee	Bayara, United Arab Emirates	4.4	GCU
		Aswan, Egypt	3.5	GCE
		Saudi Arabia	1.3	GCS
		Alameed coffee, Kuwait	5.1	GCK

*: Relative to green coffee seed, based on melanoidin content measured using a UV spectrophotometer set at 200–450 nm. **: Unavailable.

**Table 2:** Metabolites identified in methanol extracts of authenticated green *C. robusta* (GCC), green *C. arabica* (GCA), roasted *C. robusta* (RCC), and roasted *C. arabica* (RCA) via UHPLC-PDA-ESI–HRMS in both negative and positive ionization modes. Annotation of detected peaks was based on previous literature, retention times, tandem MS, and molecular networking.

Peak No.	Metabolite	R_t_ (Min)	UV Max (nm)	Mass Error (ppm)	Mol. Formula	[M − H]^−^ (*m/z*)	[M + H]^+^ (*m/z*)	MS^n^ Fragmentation Ions (*m/z*)	Method of Annotation	Coffee Specimen
	**Organic acids**									
P1	Quinic acid *	0.67	265	−0.11	C_7_H_11_O_6_^−^	191.05589	**	111, 173	[24]	RCA RCC GCA GCC
P2	Isocitric acid	0.89	372	−0.13	C_6_H_7_O_7_^−^	191.01965	**	111, 173, 155, 127		RCA RCC GCA GCC
	**Phenolic acid glycosides**									
P3	Unknown phenolic acid glycoside	0.66	265	7.92	C_17_H_17_O_10_^+^	**	381.07895	219, 201		RCA RCC GCA GCC
P4	Dihydroferulic acid 4-*O*-glucuronide	0.94	**	−1.11	C_16_H_19_O_10_^−^	371.09756	**	353, 191, 135	[25]	GCC
	**Alkaloids**									
P5	Trigonelline *	1.37	**	−1.0	C_7_H_7_NO_2_^+^	**	138.08978	120, 110, 69, 90	[26]	RCA RCC GCA GCC
P6	Caffeine *	3.54	**	−5.4	C_8_H_10_N_4_O_2_^+^	**	195.19037	137		RCA RCC GCA GCC
	**Hydroxycinnamate esters and lactones**									
P7	3-*O*-caffeoylquinic acid *	1.9	221, 325	−0.1	C_16_H_17_O_9_^−^	353.08777	**	191, 179, 135	[27,28]	RCA RCC GCA GCC
P8	5-*O*-caffeoylquinic acid *	3.69	221, 325	−1.06	C_16_H_17_O_9_^−^	353.08771	**	191, 179		RCA RCC GCA GCC
P9	4-*O*-caffeoylquinic acid	5.09	221,325	−1.06	C_16_H_17_O_9_^−^	353.08844	**	173, 179, 191		RCA RCC GCA GCC
P10	Caffeoyl shikimic acid	6.15	301,284	−2.18	C_16_H_15_O_8_^−^	335.07687	**	179, 161, 135	[29]	RCA RCC
P11	Feruloyl quinic acid isomer	7.66	221,325	−1.07	C_17_H_19_O_9_^−^	367.10324	**	161, 193, 135	[29,30]	RCA RCC GCA GCC
P12	*p*-Coumaroyl quinic acid	7.86	221,325	−1.69	C_16_H_17_O_8_^−^	337.09256	**	191, 163		RCA RCC GCA GCC
P13	Caffeoyl-quinolactone	8.99	221,325	−1.55	C_16_H_15_O_8_^−^	335.07648	**	161, 135, 179	[29]	RCA RCC
P14	Unknown chlorogenic acid derivative	9.66	218,322	−0.76	C_24_H_25_O_11_^−^	489.13986	**	353, 315, 255, 191, 297	****	RCC
P15	Unknown diacyl chlorogenic acid derivative	9.7	220,320	−2.3	C_21_H_25_O_11_^−^	453.13919	**	353, 335, 291	****	GCC
P16	Methyl-*O*-feruloyl quinic acid	10.01	218,326	−2.06	C_18_H_21_O_9_^−^	381.11981	**	175, 160, 193	[31]	RCC RCA
P17	Feruloyl-quinolactone	10.15	218,326	−0.51	C_17_H_17_O_8_^−^	349.09271	**	175, 193, 149, 134	[32]	RCC RCA
P18	Dicaffeoyl-quinolactone **	10.19	325	−0.27	C_25_H_23_O_11_^−^	497.1075	**	335	****	RCA RCC
P19	Dicaffeoyl quinic acid *	10.23	220,325	−1.76	C_25_H_23_O_12_^−^	515.11859	**	353, 335	[30]	RCA RCC GCA GCC
P20	Caffeoyl-feruloylquinic acid	10.82	325	−1.75	C_26_H_25_O_12_^−^	529.13428	**	367, 353	[25,31]	RCA RCC GCA GCC
P21	Caffeoyl-feruloyl quinolactone ***	11.74	220,325	−1.8	C_26_H_23_O_11_^−^	511.12366	**	335, 179.161	[31]	RCC
P22	Sinapoyl-feruoylquinic acid	11.28	221,324	−1.57	C_28_H_29_O_13_^−^	573.16046	**	349, 397		GCA GCC
P23	Di-feruloylquinic acid	11.31	221,324	−1.87	C_27_H_27_O_12_^−^	543.1499	**	367, 349	[25,33,34]	RCC GCC
P24	Caffeoyl-dimethoxy cinnamoylquinic acid	11.30	222,324	−1.57	C_27_H_27_O_12_^−^	543.1500	**	381.367, 335		GCA
P25	Unknown quinolactone derivative	11.6	221	−2.37	C_25_H_21_O_10_^−^	481.11288	**	335, 179, 161	****	RCC RCA
P26	Feruloyl -dimethoxycinnamoylquinic acid	11.75	222	1.62	C_28_H_29_O_12_^−^	557.1684	**	381, 349	[25,33,34]	GCA GCC
P27	Triacyl-*O*-caffeoyl-*O*-feruloyl-*O*-sinapoylquinic acid ***	11.9	222	−1.91	C_37_H_35_O_16_^−^	735.19165	**	573, 529	[31,33]	GCC
P28	Di-*O*-feruolyl-*O*-caffeoylquinic acid	11.98	222	−3.9	C_36_H_33_O_15_^−^	705.17969	**	543, 529		GCC
P29	Caffeoyl-feruloylquinic acid lactone	12.1	222	−2.13	C_26_H_23_O_11_^−^	437.14474	**	335, 193, 179	****	RCC
P30	Unknown quinolactone derivative	12.23	223	−2.8	C_21_H_23_O_9_^−^	419.13358	**	335, 317, 255, 179		RCA RCC
P31	Unknown chlorogenic acid	13.15	222	−1.23	C_21_H_25_O_10_^−^	437.1444	**	173, 275		GCA GCC
	**Sugars and sugar derivatives**									
P32	Di-*O*-hexoside	0.7	**	−0.3	C_12_H_21_O_11_^−^	341.10883	**	**		GCA GCC
P33	Acetyl-diferuloyl sucrose	8.85	221,325	−1.01	C_34_H_39_O_18_^−^	735.21344	**	367	[35,36]	GCC GCA
P34	Acyl sucroses dihydroxycinnamoyl	9.73	220,327	−0.34	C_29_H_36_O_18_^−^	671.29065	**	627		GCA
	**Diterpenes**									
P35	Cafestol	9.39	222	−1.54	C_20_H_29_O_3_^+^	**	317.21063	299, 271, 253	[37]	GCA
P36	Trihydroxy-kauradienolide ***	9.45	217	−2.1	C_20_H_27_O_5_^+^	**	347.18457	329, 285	[38]	GCA
P37	Dehydrocafestol	9.49	220	−1.32	C_20_H_27_O_2_^+^	**	299.20016	145, 191, 281, 253	[37]	GCA RCA
P38	Mozambioside	10.03	298	−1.38	C_26_H_37_O_10_^+^	**	509.224	347, 329, 311	[39,40]	RCA GCA
P39	Bengalensol-*O*-hexoside	11.75	221	−1.38	C_26_H_35_O_9_^+^	**	491.2417	329, 311	****	RCA
P40	Trihydroxy-kauranoic acid	10.67	220	−2.21	C_20_H_31_O_5_^−^	351.2171	**	289, 321	[38]	RCA
P41	Bengalensol	11.63	221	−1.35	C_20_H_25_O_4_^+^	**	329.17429	293, 311, 237	****	RCA
P42	Dihydroxy-kauren-oic acid	12.64	223	−0.218	C_20_H_29_O_4_^−^	333.20706	**	303		RCA
P43	16-methyl kahweol	12.78	222	−1.79	C_20_H_27_O_4_^+^	**	331.19006	314, 296, 145, 279	[37]	RCC RCA
P44	Dehydro-kahweol	13.6	222	−1.33	C_20_H_25_O_2_^+^	**	297.18451	279, 145		GCA
P45	Dehydrocafestol derivative	15.28	225	−1.62	C_20_H_25_O^+^	**	281.18954	263, 173, 131	[41]	RCA RCC
P46	Carboxyatractyligenin-*O*-hexoside	9.57	324,221	−1.74	C_26_H_37_O_11_^−^	525.23322	**	396, 203		GCA GCC
P47	Atracyligenin-*O*-hexoside	9.82	219,311	−1.1	C_25_H_37_O_9_^−^	481.24377	**	301	[34]	GCA GCC
P48	Desoxycarboxyatractyligenin-*O*-hexoside	10.5	220	0.05	C_37_H_55_O_17_^−^	771.34338	**	727	****	GCA
P49	Desoxyatractyligenin-*O*-hexoside	11.21	**	0.45	C_36_H_55_O_15_^−^	727.35376	**	643, 625		RCA GCA
P50	Carboxyatractyligenin-*O*-hexoside	11.72	221	−1.64	C_31_H_45_O_12_^−^	609.29065	**	565		GCA
P51	Isovaleryl- atractyligenin-*O*-hexoside derivative	11.82	221	−1.23	C_30_H_45_O_10_^−^	565.3009	**	481, 463, 303		RCA GCA
	**Fatty acids and sphingolipids**									
P52	Trihydroxy-octadecaenoic acid	12.2	223	−1.87	C_18_H_33_O_5_^−^	329.23273	**	311, 293, 229, 171	[11,42]	RCA GCA
P53	Hexosyl-2-(pentanoyloxy) propyl dodecenoate	13.03	223	−1.29	C_26_H_45_O_10_^−^	517.302	**	473, 367		RCC
P54	Linoleic acid methyl ester ***	13.19	223	−1.47	C_17_H_25_O_4_^−^	293.17523	**	236, 221	[11]	RCC GCC
P55	Unknown fatty acid	13.6	222	4.18	C_14_H_29_O_8_^−^	325.18228	**	183		RCA RCC GCA GCC
P56	Sphingolipid conjugate I ***	14.00	222	1.39	C_27_H_49_NO_9_P^−^	562.31610	**	502	****	RCA RCC GCA GCC
P57	Sphingolipid conjugate II ***	14.55	222	−2.18	C_27_H_51_NO_9_P^−^	564.32947	**	504		RCA RCC GCA GCC
P58	Phosphatidyl inositol hexanoic acid derivative	14.68	224	−3.21	C_25_H_48_O_12_P^−^	571.28705	**	391, 315, 255, 241	[11]	RCA RCC GCA GCC
P59	Ceramide conjugate I ***	14.75	224	−0.45	C_22_H_49_O_6_N_4_P^+^	**	496.33881	478, 184		RCA RCC GCA GCC
P60	Sphingolipid conjugate III ***	14.78	222	−1.9	C_25_H_51_NO_9_P^−^	540.32941	**	480	****	RCA RCC GCA GCC
P61	Unknown fatty acid	14.85	224	−7.34	C_13_H_27_O_8_^−^	311.16809	**	183		RCA RCC GCA GCC
P62	Sphingolipid conjugate IV ***	14.97	224	−1.45	C_27_H_53_NO_9_P^−^	566.34552	**	506	****	RCA RCC GCA GCC
P63	Ceramide conjugate II	15.08	221	−0.98	C_21_H_45_O_2_N_9_P_2_^+^	**	522.35461	504, 184		RCA RCC GCA GCC
P64	Unknown fatty acid ester	15.48	222	8.46	C_24_H_51_O_10_^−^	499.35391	**	481, 455, 322, 279		RCA RCC GCA GCC
P65	Ceramide conjugate III ***	15.7	222	−1.42	C_23_ H_46_O_8_ N_5_^+^	**	520.37042	502, 184		RCC GCC GCC GCA
P66	Unknown hydroxy fatty acid	16.22	225	−8.13	C_28_H_31_O_3_^−^	415.22482	**	279		RCC
P67	Unknown fatty acid	16.3	225	−8.08	C_26_H_55_O_10_^−^	527.38416	**	509, 350, 307		RCC RCA
P68	Unknown fatty acid	16.49	225	−9.23	C_15_H_31_O_8_^−^	339.19919	**	183		RCA RCC GCA GCC
P69	Dimethyl octadecanedioate	19.18	226	0.83	C_20_H_37_O_4_^−^	341.26892	**	313, 269	[11]	RCA RCC GCA GCC
P70	Hydroxy-docosanoic acid	19.35	224	−2.97	C_22_H_43_O_3_^−^	355.32135	**	309		RCA RCC GCA GCC
P71	Hydroxy-tetracosanoic acid	20.58	222	−2.5	C_24_H_47_O_3_^−^	383.35208	**	337		RCA RCC GCA GCC
P72	Unknown fatty acid ester	20.97	227	−0.03	C_38_H_55_O_3_^−^	559.41382	**	541, 279, 223, 183		RCC GCC
	**Fatty acyl amides**									
P73	Unknown fatty acid amide	15.77	224	−0.06	C_20_H_38_O_2_N^+^	**	324.28943	307, 263, 245		RCC RCA
P74	Docosenamide ***	16.69	226	−1.06	C_22_H_44_NO^+^	**	338.34137	321,303	[43]	RCA RCC GCC GCA
	**Nitrogenous compounds (hydroxytryptamine derivatives)**									
P75	*N*-heneicosanoyl- hydroxytryptamine	15.92	226	−1.4	C_30_H_51_N_2_ O_3_^+^	**	487.39	469, 177, 160		RCA GCA RCC GCC
P76	*N*-tricosanoyl-hydroxytryptamine	16.69	226	−0.65	C_23_H_55_N_2_O_3_^+^	**	515.42	497, 177, 160	[44,45]	RCA RCC GCA GCC
P77	*N*-docosanoyl-hydroxytryptamide	19.3	226	−1.09	C_32_H_55_N_2_O_5_^+^	**	499.42	482, 177, 160		RCA RCC GCA GCC
P78	*N*-octadecanoyl-5-hydroxytryptamide	17.46	226	−1.81	C_28_H_46_N_2_O_2_^+^	**	443.36185	426, 177, 160		RCA RCC GCA GCC
P79	*N*-eicosanoyl-hydroxytryptamide	18.32	226	−1.68	C_30_H_51_N_2_O_2_^+^	**	471.39349	454, 177, 160		RCA RCC GCA GCC
P80	*N*-tetracosanoyl-hydroxytryptamide	20.32	226	−0.95	C_34_H_59_N_2_O_2_^+^	**	527.46	510,177,160		RCA RCC GCA GCC
	**Hydroxycinnamoyl amides**									
P81	Unknown caffeoyl amide	9.8	220	9.75	C_18_H_16_NO_6_^−^	342.09775	**	206	[25]	GCA GCC
P82	Caffeoyl-*N*- tryptophan	11.08	221	−0.61	C_20_H_17_N_2_O_5_^−^	365.11395	**	135,229	[25,46]	GCC RCC
P83	*p*-Coumaroyl-*N*-tryptophan	11.49	221	−2.0	C_20_H_17_N_2_O_4_^−^	349.11868	**	229		RCC GCC
P84	Unknown feruloyl amide	11.66	222	9.14	C_21_H_19_N_2_O_5_^−^	379.12805	**	335, 229		GCC
P85	Unknown hydroxy cinnamic acid amide	12.1	229	−6.59	C_25_H_21_O_6_N_2_^−^	445.1405	**	309, 161, 229	****	RCA RCC
	**Unknowns**									
P86	Unknown	9.69	222	−1.07	C_19_H_25_O_2_^+^	**	285.18472	267, 239, 229		RCA
P87		11.81	222	−0.64	C_22_H_27_O_10_^−^	451.15973	**	349, 275, 173		GCA GCC
P88		15.3	222	−2.3	C_28_H_33_O_4_^−^	433.23483	**	153		GCA

*: identified by comparison with authentic standard. **: not detected. ***: reported for the first time. ****: Global Natural Product Social Molecular Networking (GNPS).

## Data Availability

Data is contained within the article and supplementary material.

## Supplementary Materials

The following are available online at https://www.mdpi.com/article/10.3390/antiox11010131/s1.
