# Supplementary material for: How Does LC/MS Compare to UV in Coffee Authentication and Determination of Antioxidant Effects? Brazilian and Middle Eastern Coffee as Case Studies
Source: Antioxidants (Basel). 2022 Jan 7;11(1):131. doi: 10.3390/antiox11010131 (PMC8773014; doi:10.3390/antiox11010131)
Supplement: Supplementary file 1 [file antioxidants-11-00131-s001.zip › antioxidants-1470390-supplementary.pdf]

Supplementary materials

How Does LC/MS Compare to UV in Coffee Authentication and Determination of Antioxidant Effects? Brazilian and Middle Eastern Coffee as Case Studies

Enas A. El-Hawary [1], Ahmed Zayed [2,3], Annegret Laub [4], Luzia V. Modolo [5], Ludger Wessjohann [4,*] and Mohamed A. Farag [6,*]

[1] Chemistry Department, School of Sciences & Engineering, The American University in Cairo, New Cairo 11835, Egypt; enaselhawary17@aucegypt.edu

[2] Pharmacognosy Department, College of Pharmacy, Tanta University, Elguish Street (Medical Campus), Tanta 31527, Egypt; ahmed.zayed1@pharm.tanta.edu.eg

[3] Institute of Bioprocess Engineering, Technical University of Kaiserslautern, Gottlieb-Daimler-Straße 49, 67663 Kaiserslautern, Germany

[4] Department of Bioorganic Chemistry, Leibniz Institute of Plant Biochemistry, Weinberg 3, 06120 Halle, Germany; Annegret.Laub@ipb-halle.de

[5] Departamento de Botânica, Instituto de Ciências Biológicas, Universidade Federal de Minas Gerais, Belo Horizonte 31270-901, Brazil; vmodolo@icb.ufmg.br

[6] Pharmacognosy Department, College of Pharmacy, Cairo University, Kasr El-Aini St., Cairo 11562, Egypt

* Correspondence: ludger.wessjohann@ipb-halle.de (L.W.); mohamed.farag@pharma.cu.edu.eg (M.A.F.)

Suppl. Figures

Suppl. Figure S1: UHPLC-ESI-HRMS base peak chromatograms of authenticated green *Coffea arabica, i.e.,* GCA, methanol extract as representatives in negative (A) and positive ion (B) modes. Major peaks are numbered corresponding to peak numbers listed in **Table 2**.

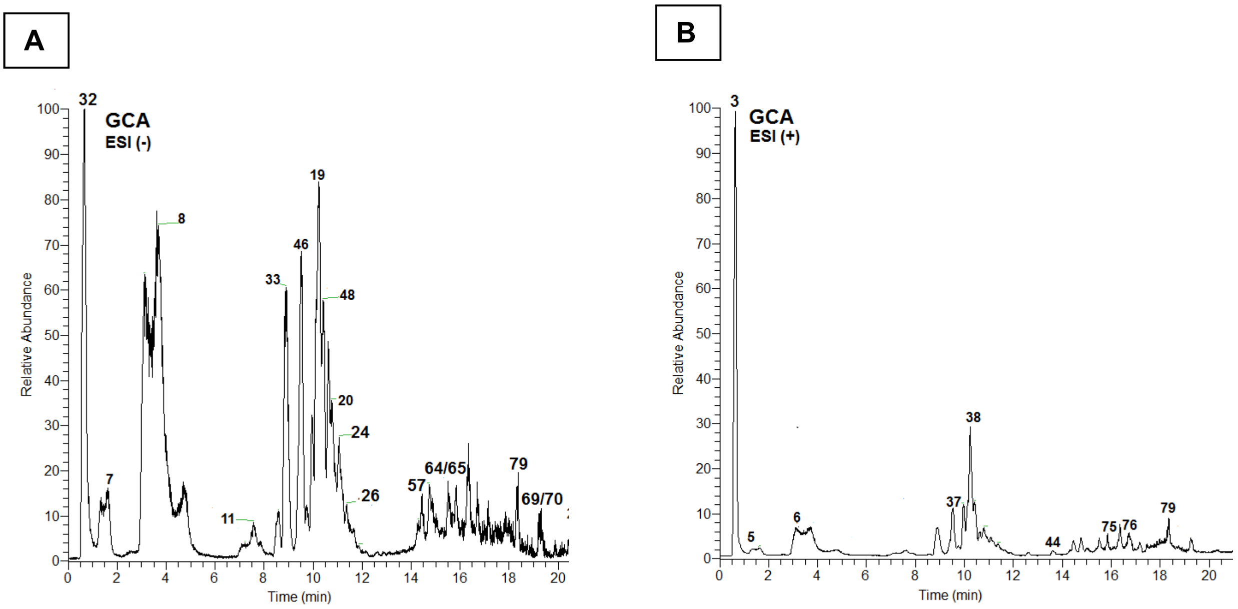

Suppl. Figure S2: MS/MS spectrum of caffeine (P6) in the positive ion mode

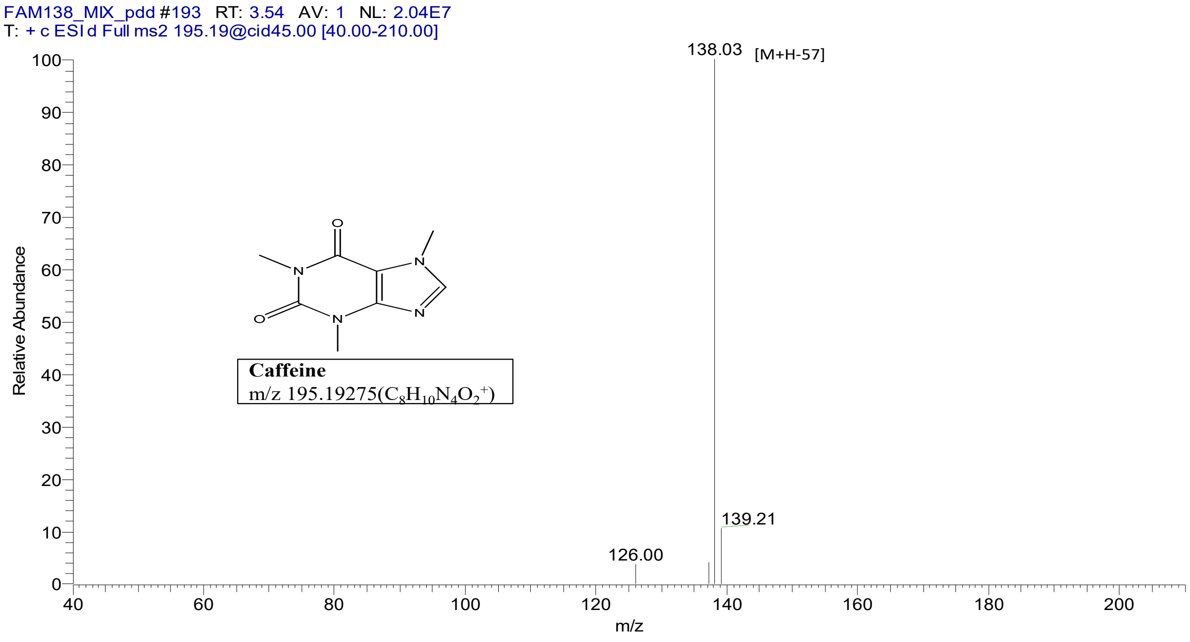

Suppl. Figure S3: MS/MS spectrum and fragmentation pattern of trigonelline (P5) in positive ion mode

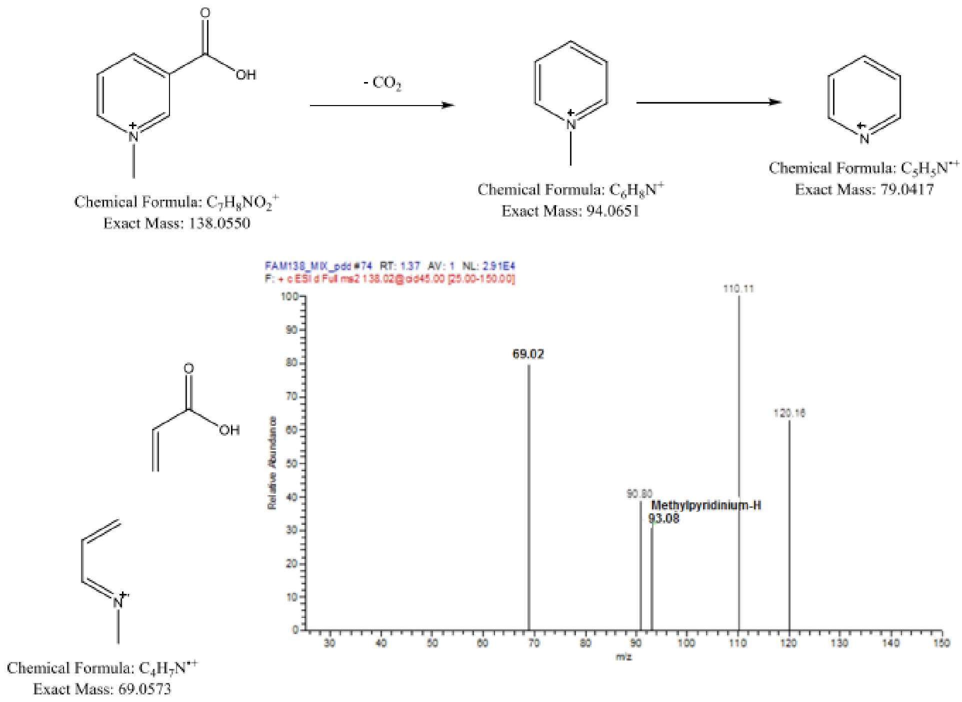

Suppl. Figure S4: MS/MS spectrum of dicaffeoylquinic acid (P19) in negative ion mode

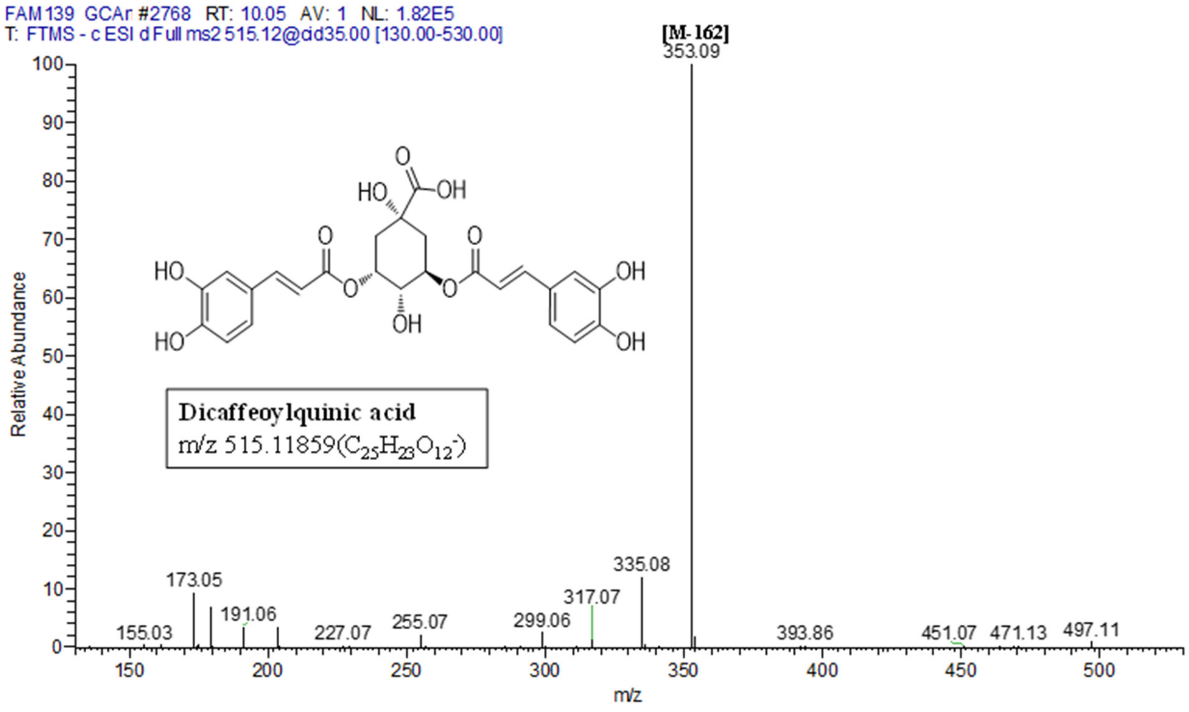

Suppl. Figure S5: MS/MS spectrum of caffeoyl-feruloylquinic acid (P20) in negative ion mode

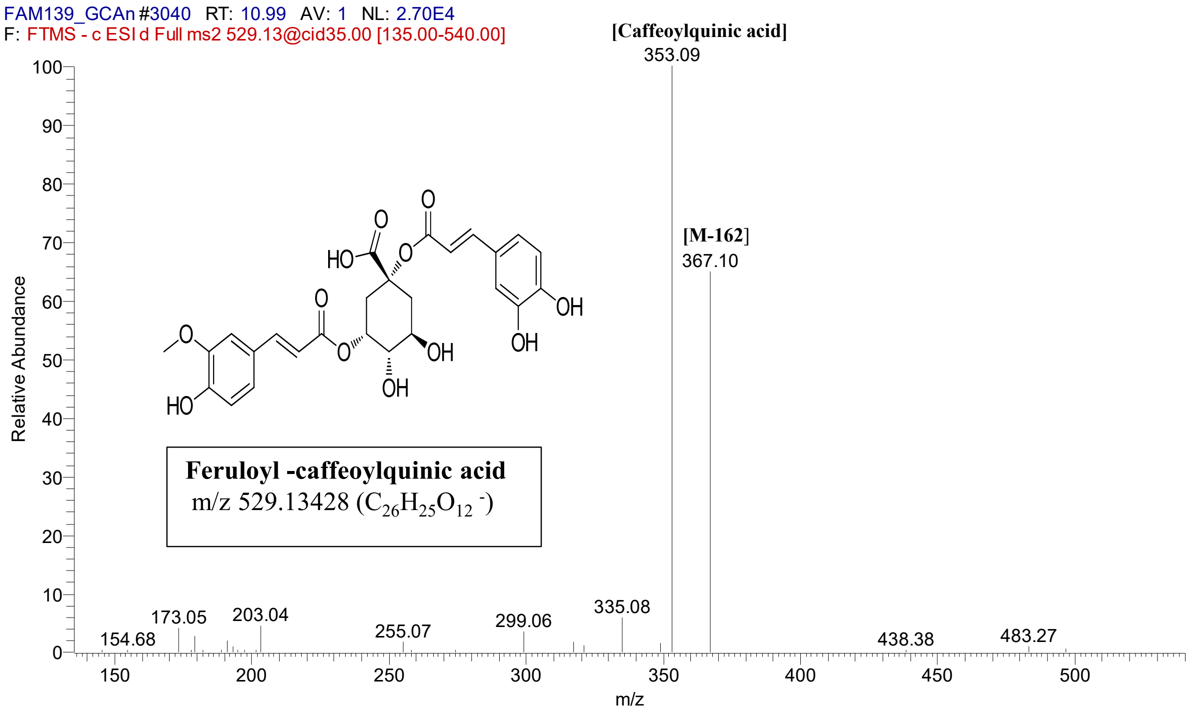

Suppl. Figure S6: MS/MS spectrum of methyl-*O*-feruloyl quinic acid (P16) in negative ion mode

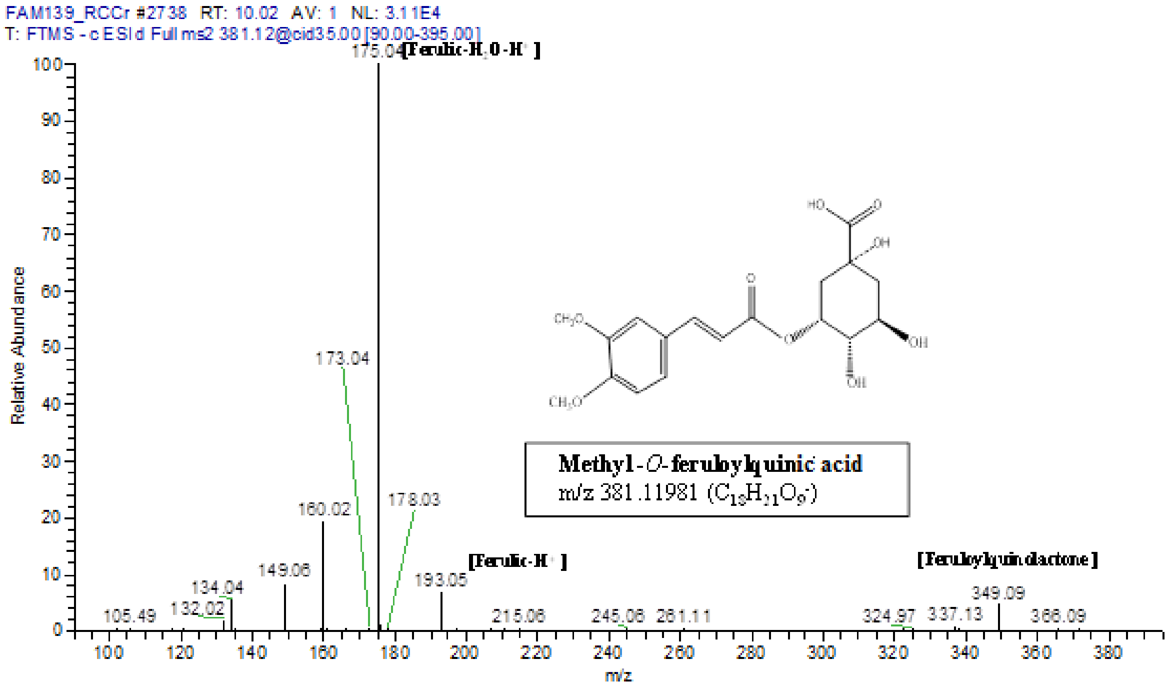

Suppl. Figure S7: MS/MS spectrum of sinapoyl-feruloyl quinic acid (P22) in negative ion mode

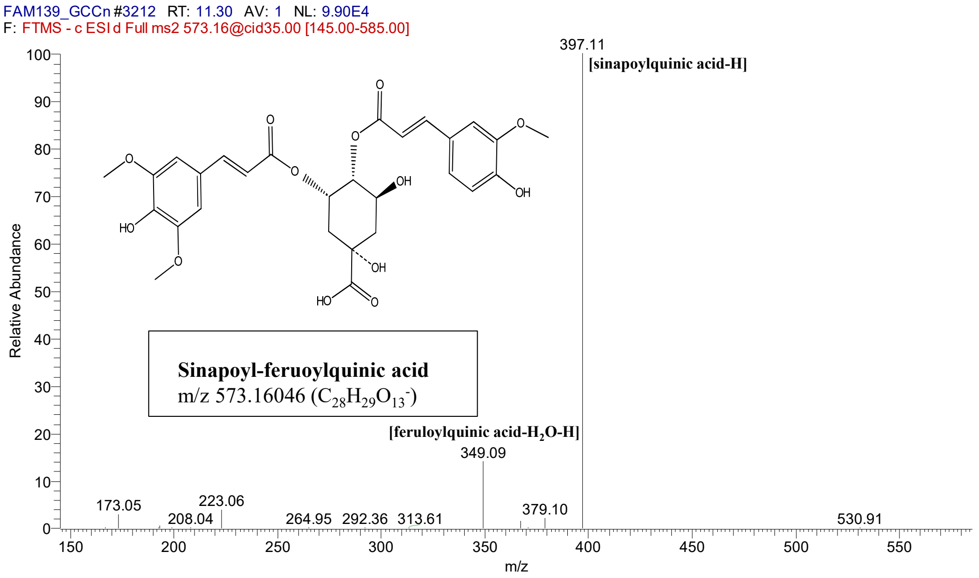

Suppl. Figure S8: MS/MS spectrum of di-*O*-feruloyl-*O*-caffeoylquinic acid (P28) in negative ion mode

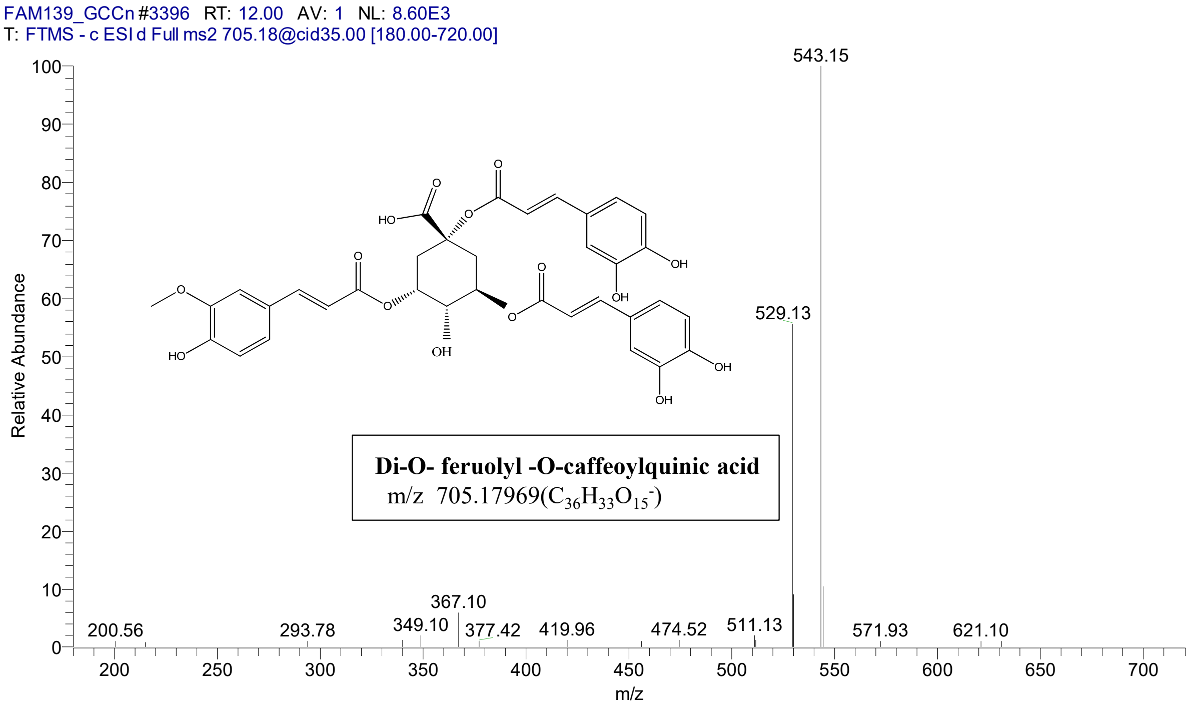

Suppl. Figure S9: MS/MS spectrum of triacyl *O*-caffeoyl-*O*-feruloyl-*O*-sinapoylquinic acid (P27) in negative ion mode

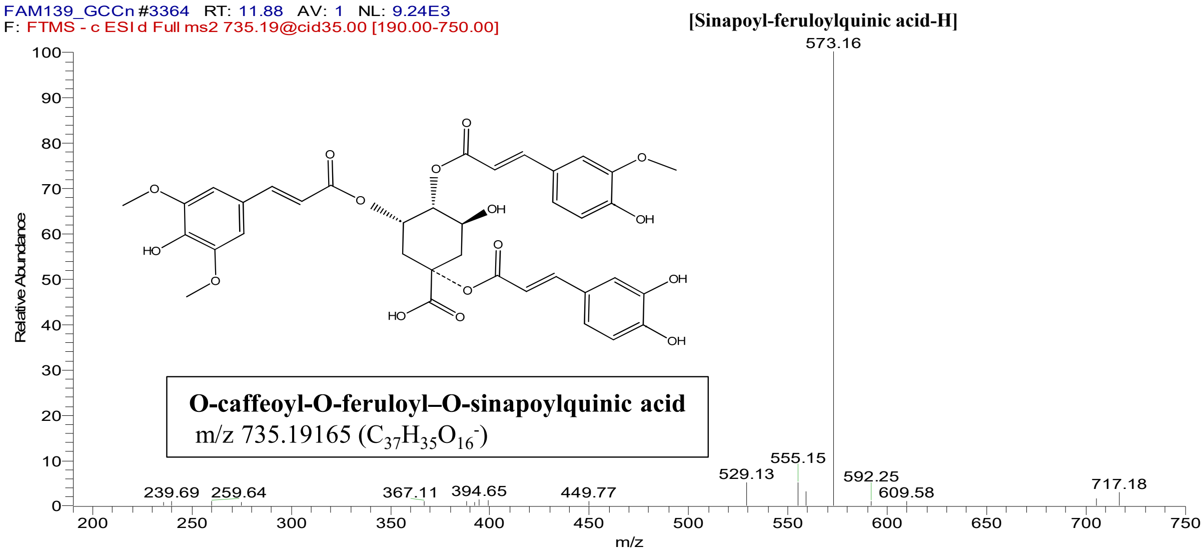

Suppl. Figure S10: MS/MS spectrum of dihydro ferulic acid-*O*-glucuronide (P4) in negative ion mode

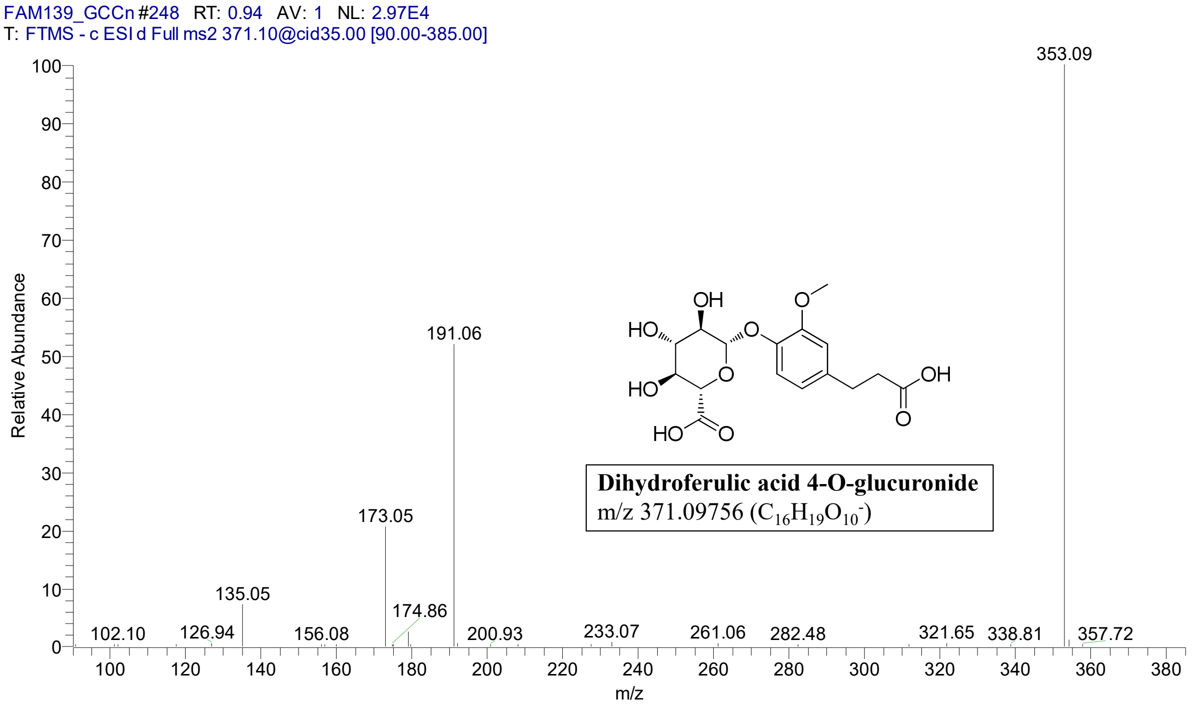

Suppl. Figure S1: MS/MS spectrum of feruloyl-quinolactone (P17) in negative ion mode

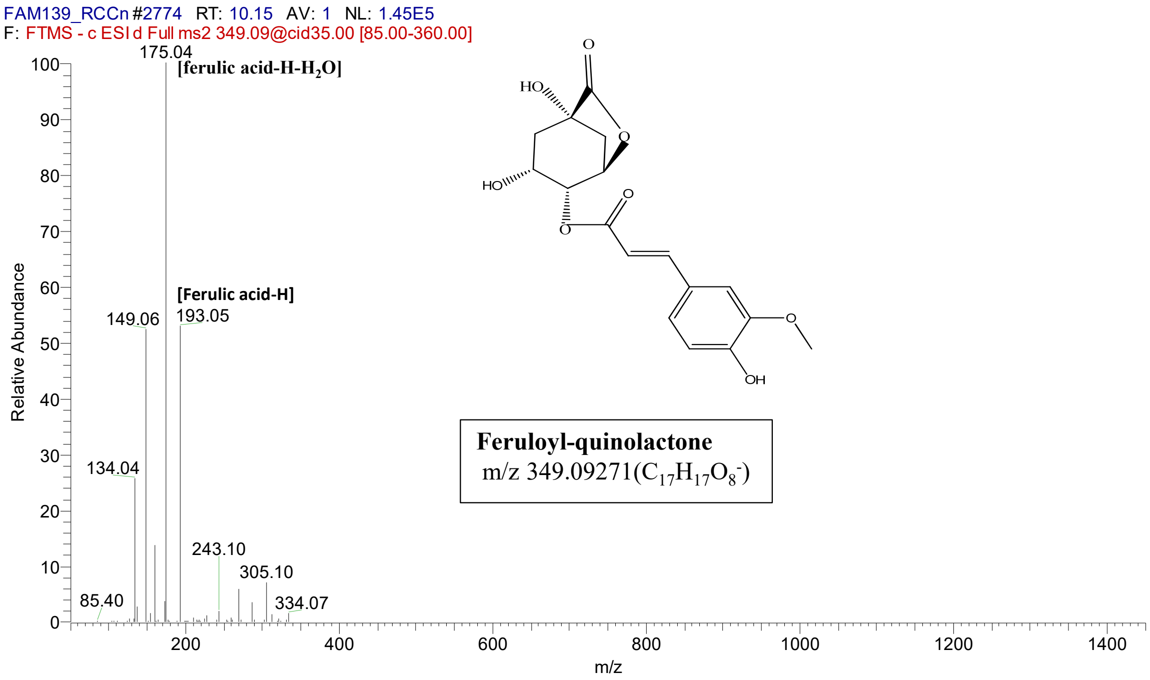

Suppl. Figure S2: MS/MS spectrum of cafestol (P35) in positive ion mode

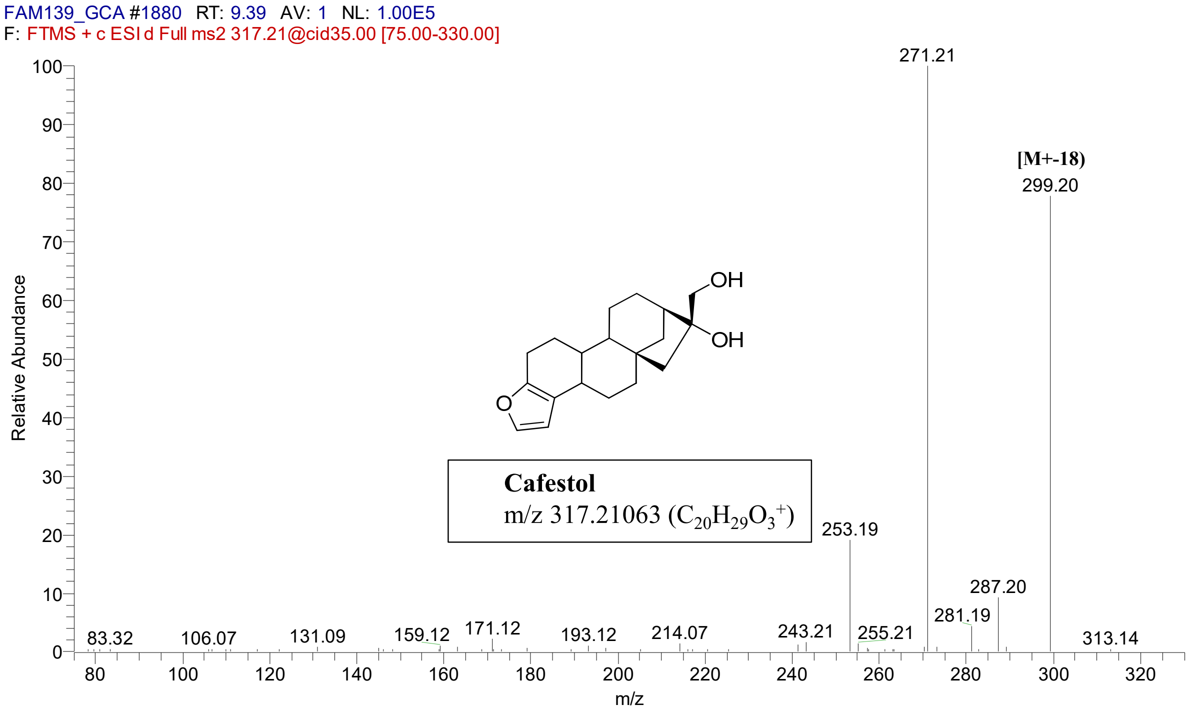

Suppl. Figure S3: MS/MS spectrum of dehydrocafestol (P37) in positive ion mode

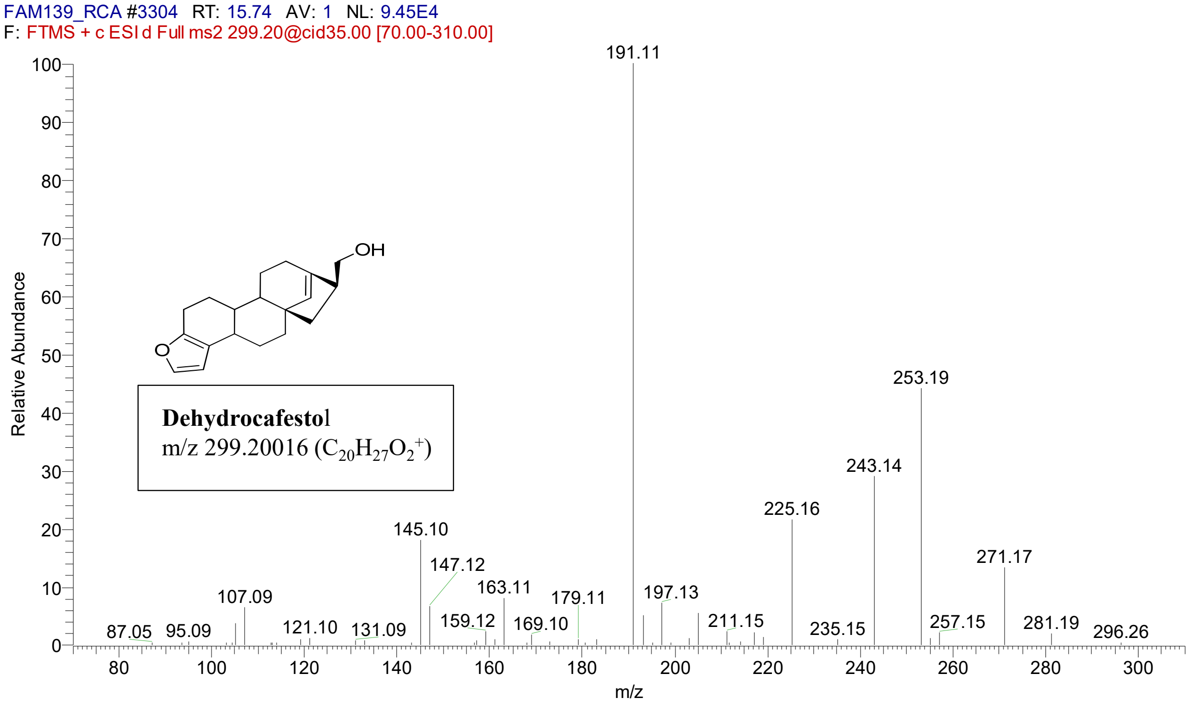

Suppl. Figure S14: MS/MS spectrum of dehydrocafestol derivatives (P45) in positive ion mode

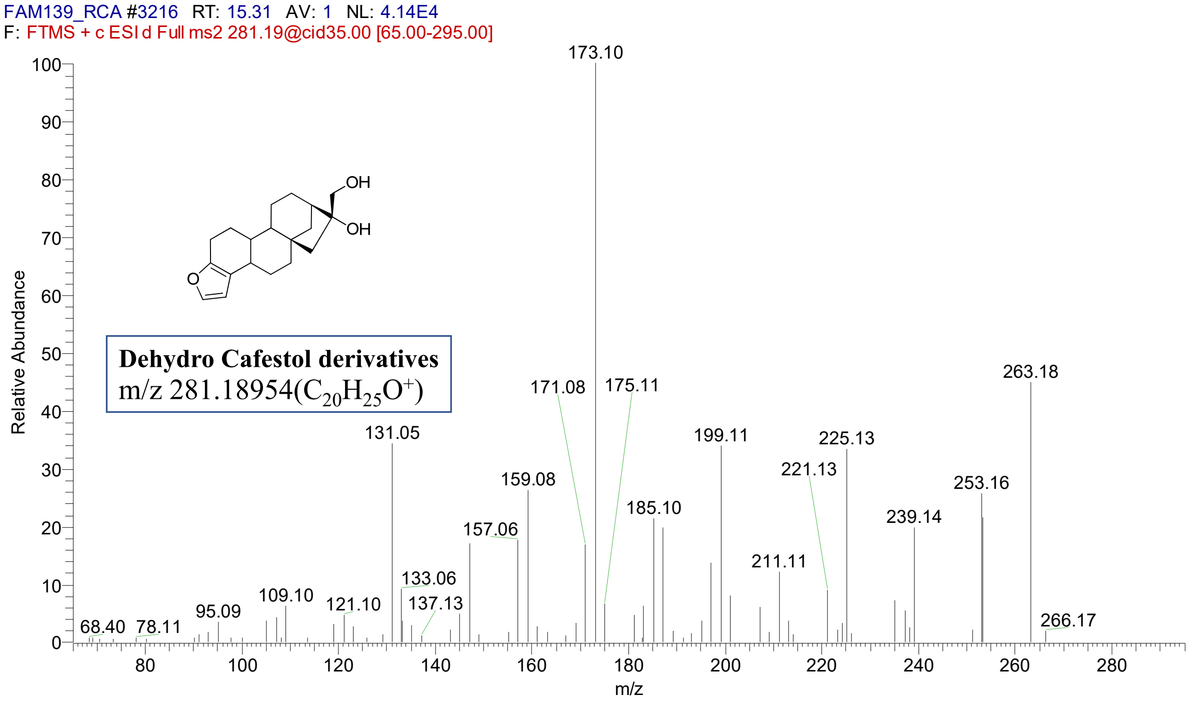

Suppl. Figure S15: MS/MS spectrum of mozambioside (P38) in positive ion mode

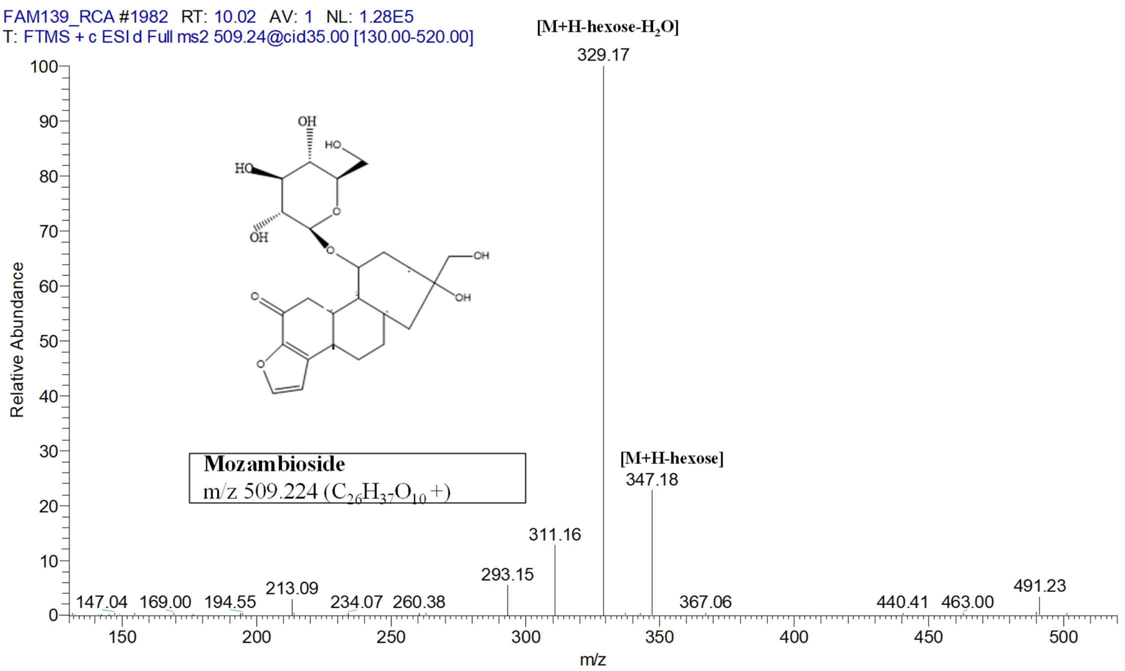

Suppl. Figure S16: MS/MS spectrum of trihydroxy-kauradien-olide (P36) in positive ion mode

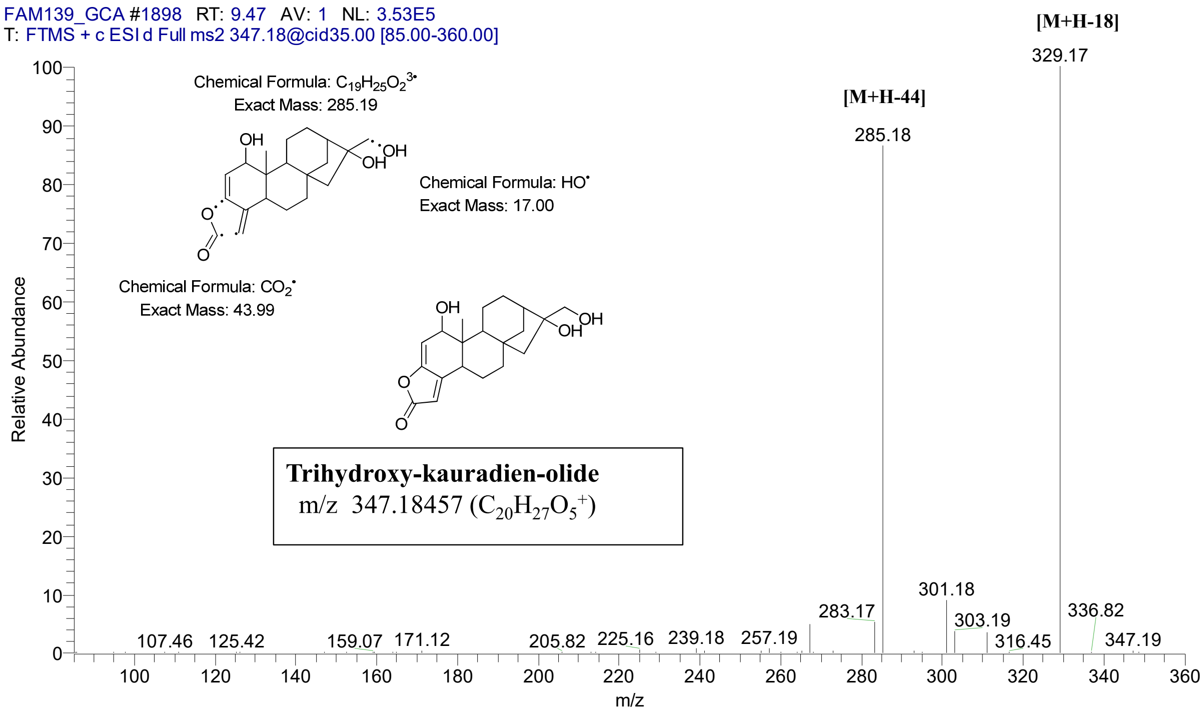

Suppl. Figure S17: MS/MS spectrum of dimethyl octadecanedioate (P70) in negative ion mode

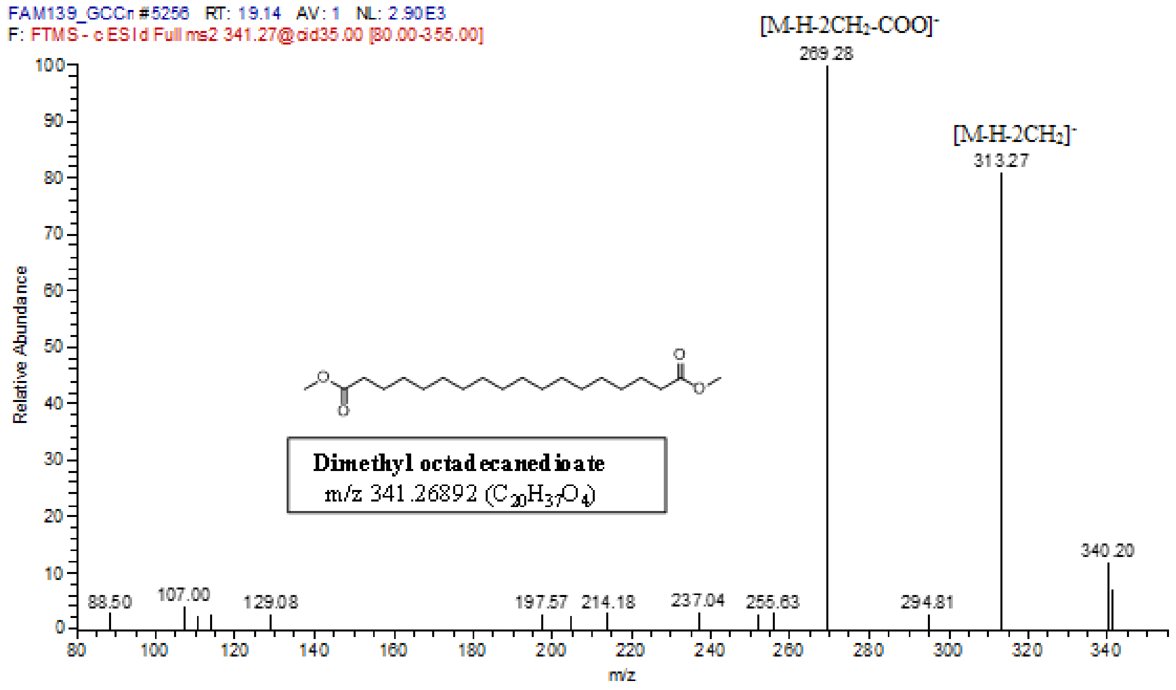

Suppl. Figure S18: MS/MS spectrum of docosenamide (P74) in positive ion mode

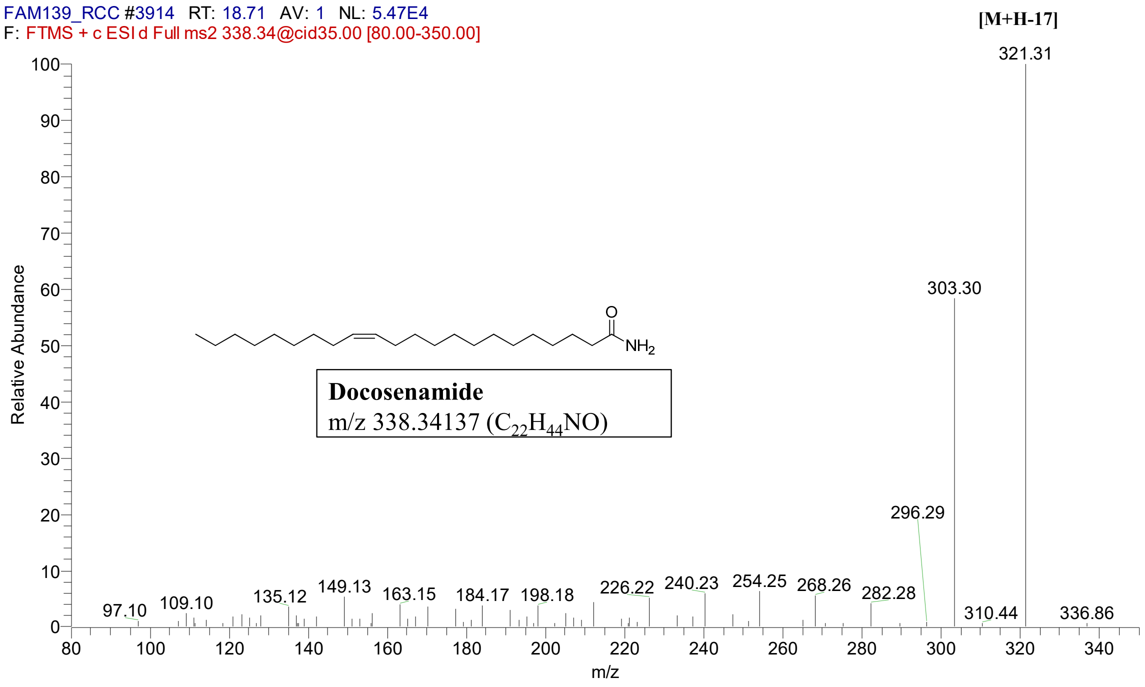

Suppl. Figure S19: MS/MS spectrum of *N*-heneicosanoyl-5-hydroxytryptamine (P75) in positive ion mode

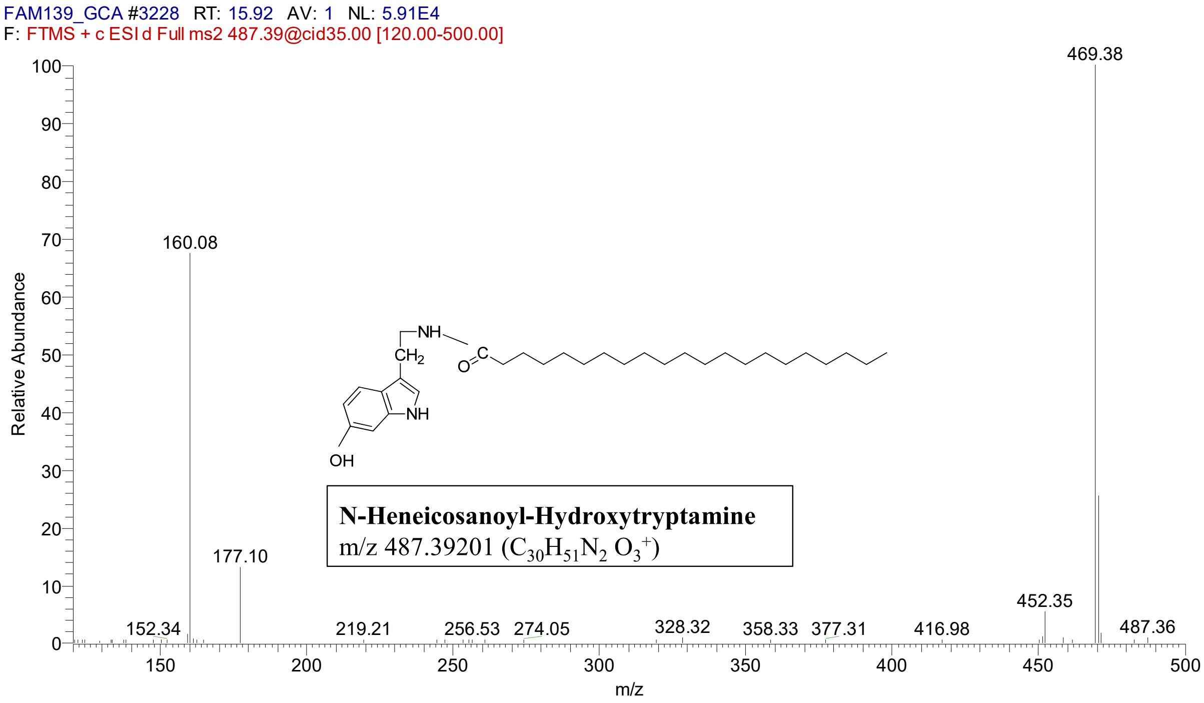

Suppl. Figure S20: MS/MS spectrum of *N*-octadecanoyl-5-hydroxytryptamide (P78) in positive ion mode

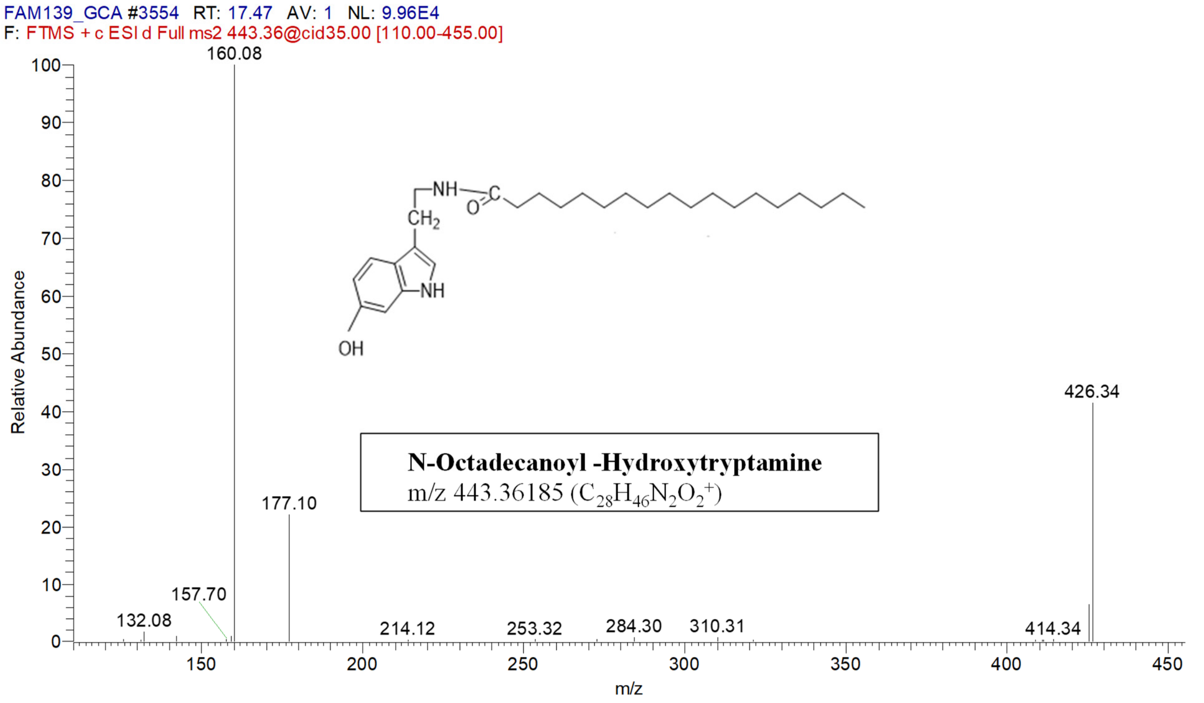

Suppl. Figure S21: MS/MS spectrum feruloyl ester of sucrose (P33) in negative ion mode

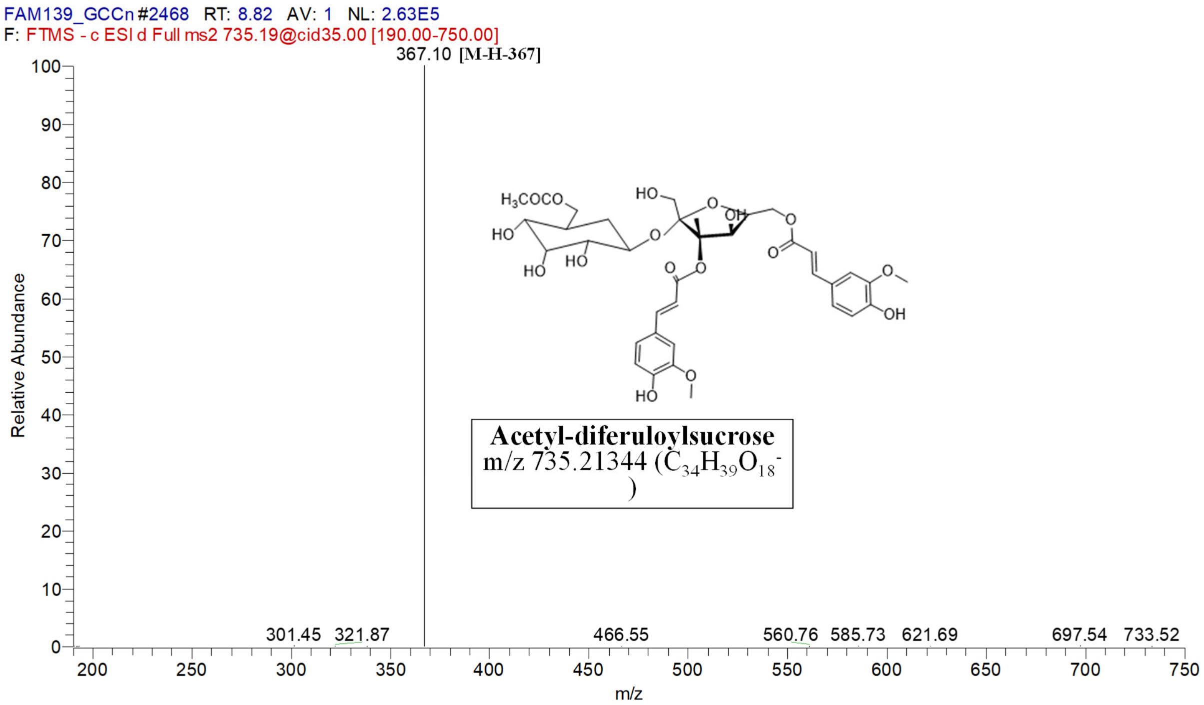

Suppl. Figure S22: Multivariate principal component analysis (PCA) for coffee specimens based on UPLC/MS analysis. **(A)** PCA score plot for green versus roasted samples, (**B)** PCA loading plot for green versus roasted samples, **(C)** PCA score plot for roasted versus instant samples, **(D)** PCA score plot for plain versus blended with cardamom samples, and **(E)** PCA loading plot for plain versus blended with cardamom samples.

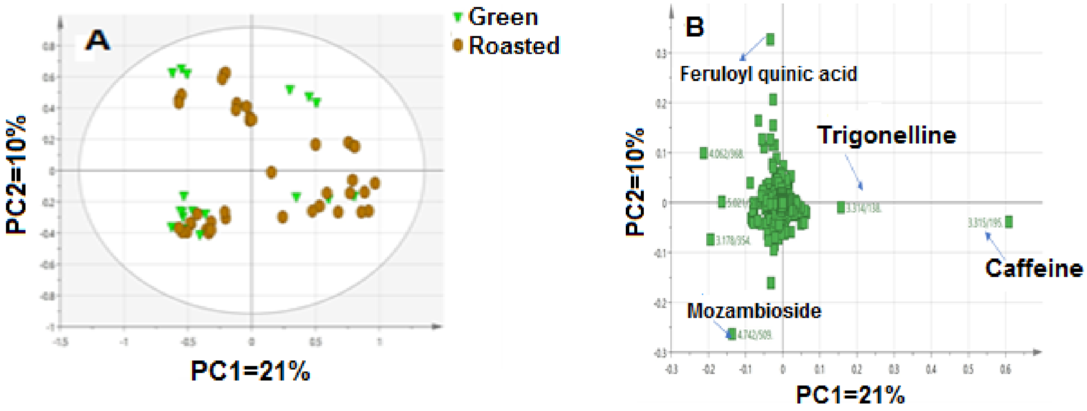

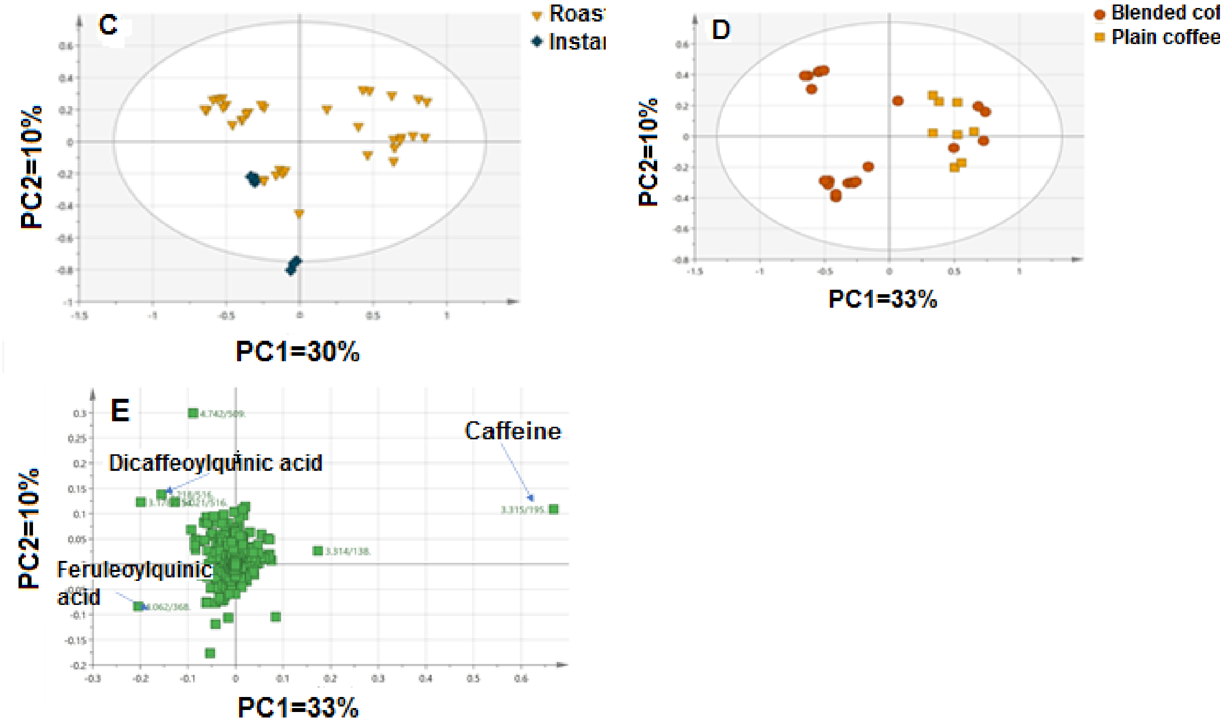

Suppl. Figure S23: OPLS-DA model validation for modelling green versus roasted coffee specimens detected using LC-MS. **(A)** the diagnostic metrics R^2 and Q^2, (**B**) permutation testing. n=20, and **(C)** CV-ANOVA to assess for model statistical significance. **(D)** SECV residuals

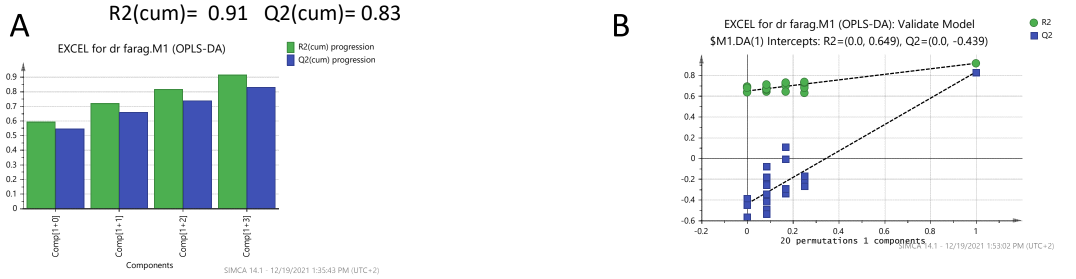

C

M1(Untitled)	SS	DF	MS	F	P value	SD
Total corr.	53	53	1			1
Regression	44.0709	8	5.50887	27.7632	5.70756e-015	2.3471
Residual	8.92906	45	0.198424			0.445448

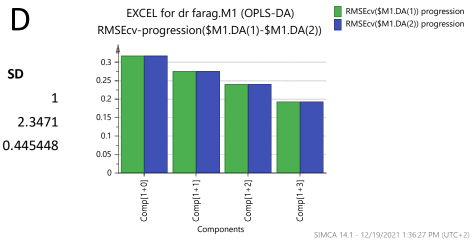

Suppl. Figure S24: OPLS-DA model validation for modelling roasted versus instant coffee specimens detected using LC-MS. **A.** the diagnostic metrics R^2 and Q^2, (**B)** permutation testing. n=20, and **(C)** CV-ANOVA to assess for model statistical significance. **(D)** SECV residuals

A

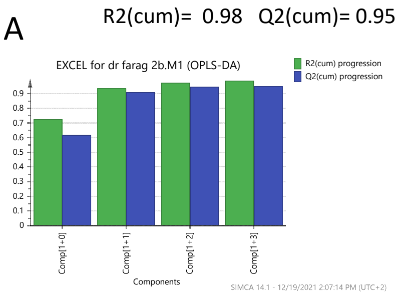

B

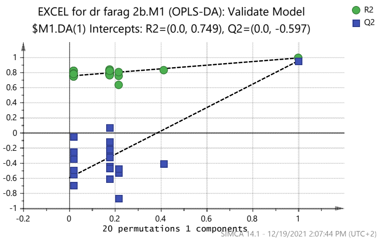

C

M1(Untitled)	SS	DF	MS	F	P value	SD
Total corr.	39	39	1			1
Regression	37.0739	8	4.63424	74.588	4.3508e-018	2.15273
Residual	1.92607	31	0.0621312			0.249261

D

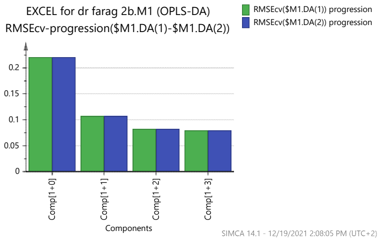

Suppl. Figure S25: OPLS-DA model validation for modelling plain versus blended coffee with cardamom samples detected using LC-MS. **(A)** the diagnostic metrics R^2 and Q^2, (**B)** permutation testing. n=20, and (**C)** CV-ANOVA to assess for model statistical significance. **(D)** SECV residuals

A

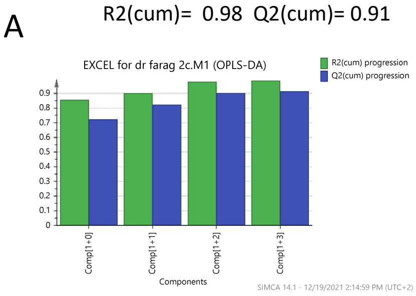

B

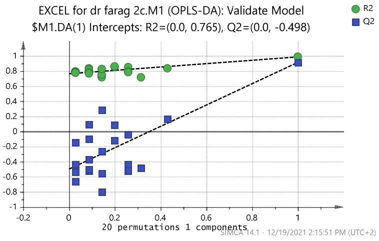

C

M1(Untitled)	SS	DF	MS	F	P value	SD
Total corr.	35	35	1			1
Regression	31.9982	8	3.99978	35.9765	1.9159e-012	1.99994
Residual	3.00179	27	0.111177			0.333433

D

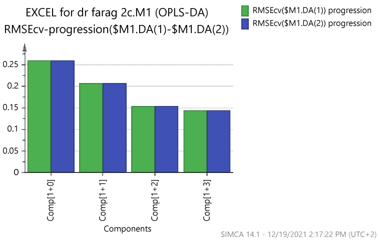

Suppl. Figure S26: Multivariate principal component analysis (PCA) score plot coffee specimens based Uv-Vis analysis. **(A)** green versus roasted samples, and **(B)** roasted versus instant coffee samples

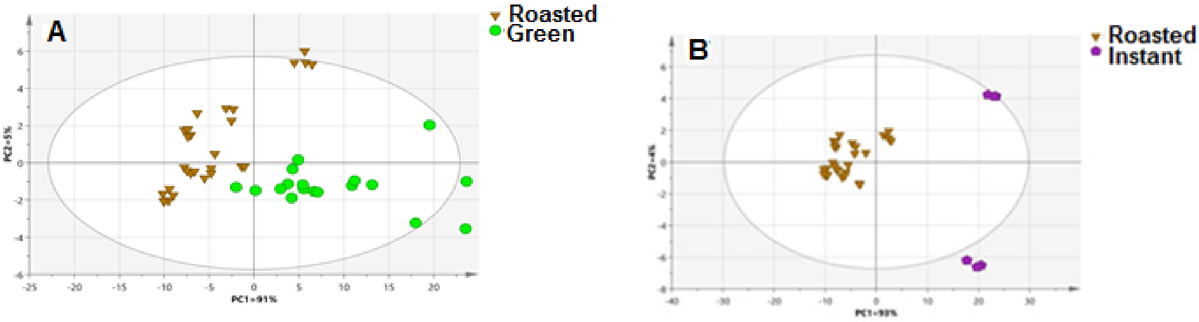

Suppl. Figure S27: Spiking with the acrylamide reference standard using UV spectrometry method. The figure shows an absorbance increase of the roasted coffee sample spiked with acrylamide at 273 nm

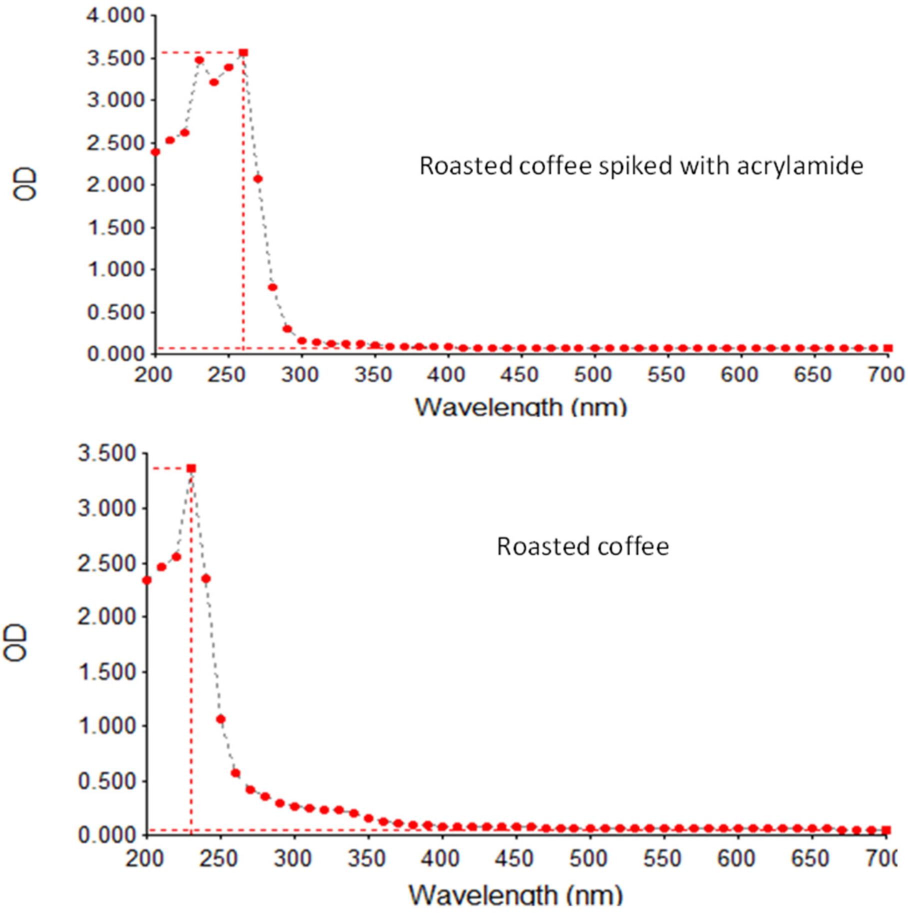

Suppl. Figure S28: Radar charts created from VIP score of the main metabolites which contributed to antioxidant activity. **(A)**: Dicaffeoyl quinolactone showed maximum concentration in roasted samples and light roasted (RCA, RCC, LRCM), respectively. **(B)**: Caffeoylquinic acid showed maximum concentration green, and light roasted (BRA, LRCM), respectively. **(C)**: Feruloylquinic acid showed maximum concentration in green, and light roasted (GCS, GCE, BRA), respectively. Samples codes are listed in **Table 1**.

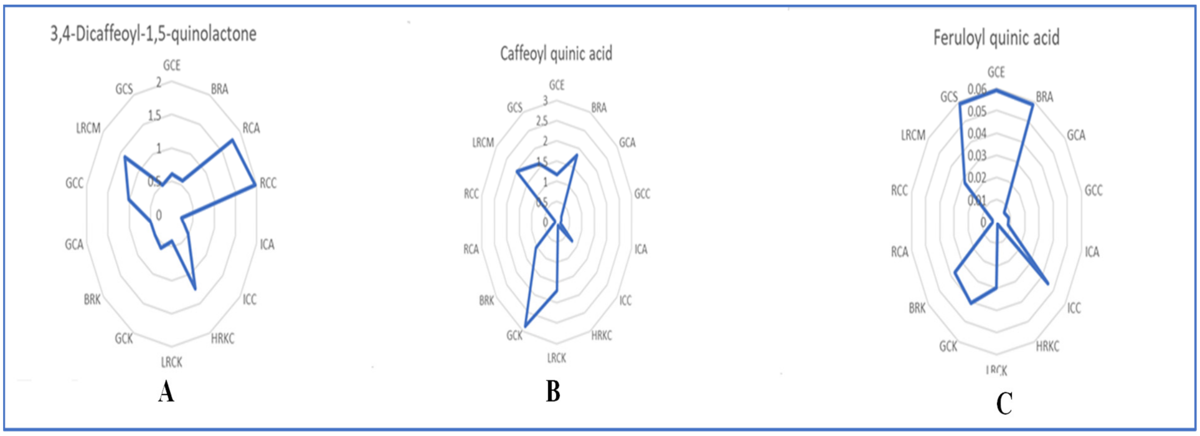

Suppl. Tables

Suppl. Table S1: Summarized results of total phenolic content and antioxidant assays in investigated coffee seeds and (TPC, DPPH, FRAP ± SD, n=3). The corresponding sample codes are listed in **Table 1**.

Coffee samples	TPC (mg GAE/g extract)	DPPH Scavenging IC_{50} (µg/mL)	FRAP (mg TE/mg extract)
BRK	18.2±0.01	27.3±2.6*	34.1±0.01*
RCA	13.6±0.01	103.3±3.2*	3.9±0.01*
RCC	15.9±0.01	74.2±1.5**	7.1±0.003*
ICA	7.7±0.01	62.5±1.9**	7.9±0.001*
LRCS	42.8±0.01	48.6±1.9*	7.1±0.002*
LRS	36.5±0.01	43.4±1.08*	9.2±0.001*
LRCK	52.3±0.01	120.9±3.1*	5.5±0.001*
LRCM	32.6±0.12	30.4±4.4*	7.9±0.002*
ICC	3.2±0.04	104.1±8.3**	1.5±0.004*
GCU	15.3±0.01	100.5±6.2*	6.3±0.002*
GCA	33.1±0.003	142±1.9*	10.3±0.001**
GCE	25.3±0.01	112.1±1.3*	15.1±0.003*
GCK	50.9±0.007	146.1±1.1*	10.3±0.001*
HRKC	36.1±0.03	234.9±5.8*	6.3±0.003*
LRCQ	14.7±0.02	187±4.1*	5.5±0.003*

GCC	46.6±0.004	66.72±2.8**	26.1±0.003 *
BRA	52.5±0.03	69.12±2.6*	28.2±0.004*
Gallic acid (mean ± SD)	100.09±0.24		
Trolox (µg/mL)		12.4±1.48*	
Trolox (mean ± SD)			1.12±0.33*

*Significant from GCC sample (*P* value < 0.05, one-way ANOVA)

**non-significant
